# Identification of modules and key genes associated with breast cancer subtypes through network analysis

**DOI:** 10.1038/s41598-024-61908-4

**Published:** 2024-05-29

**Authors:** María Daniela Mares-Quiñones, Edgardo Galán-Vásquez, Ernesto Pérez-Rueda, D. Guillermo Pérez-Ishiwara, María Olivia Medel-Flores, María del Consuelo Gómez-García

**Affiliations:** 1 Laboratorio de Biomedicina Molecular, Programa de Doctorado en Biotecnología, Escuela Nacional de Medicina y Homeopatía, Instituto Politécnico Nacional, Ciudad de México, Mexico; 2 Departamento de Ingeniería de Sistemas Computacionales y Automatización, Instituto de Investigaciones en Matemáticas Aplicadas y en Sistemas, Universidad Nacional Autónoma de México, Ciudad Universitaria, Ciudad de México, Mexico; 3 Instituto de Investigaciones en Matemáticas Aplicadas y en Sistemas, Universidad Nacional Autónoma de México, Unidad Académica del Estado de Yucatán, Mérida, Mexico

**Keywords:** Cancer, Computational biology and bioinformatics

## Abstract

Breast cancer is the most common malignancy in women around the world. Intratumor and intertumoral heterogeneity persist in mammary tumors. Therefore, the identification of biomarkers is essential for the treatment of this malignancy. This study analyzed 28,143 genes expressed in 49 breast cancer cell lines using a Weighted Gene Co-expression Network Analysis to determine specific target proteins for Basal A, Basal B, Luminal A, Luminal B, and HER2 ampl breast cancer subtypes. Sixty-five modules were identified, of which five were characterized as having a high correlation with breast cancer subtypes. Genes overexpressed in the tumor were found to participate in the following mechanisms: regulation of the apoptotic process, transcriptional regulation, angiogenesis, signaling, and cellular survival. In particular, we identified the following genes, considered as hubs: *IFIT3*, an inhibitor of viral and cellular processes; *ETS1*, a transcription factor involved in cell death and tumorigenesis; ENSG00000259723 lncRNA, expressed in cancers; AL033519.3, a hypothetical gene; and *TMEM86A*, important for regulating keratinocyte membrane properties, considered as a key in Basal A, Basal B, Luminal A, Luminal B, and HER2 ampl breast cancer subtypes, respectively. The modules and genes identified in this work can be used to identify possible biomarkers or therapeutic targets in different breast cancer subtypes.

## Introduction

Breast cancer (BC) is the most common malignancy in women and the second leading cause of cancer-related deaths in developed and industrialized countries. In 2020, there were 2.3 million new cases and 685,000 deaths worldwide^1^. BC is a process characterized by uncontrolled cell proliferation in the cells of the mammary glands, in which mutations are progressively acquired, generating genomic instability^2^.

The two most common types of BC by origin are ductal and lobular^3^. However, great morphological and biological heterogeneity generates Intrinsic Subtypes of Breast Cancer (ISBC) such as Luminal A and Luminal B, Basal-like, low in claudin, over-expressed with HER2, triple-negative, and normal type^4^. Intrinsic surrogate subtypes are also classified into triple-negative, over-expressed with HER2, Luminal B as HER2+, Luminal B as HER2−, and Luminal A^4^. Intrinsic surrogate subtypes are also classified into triple-negative, over-expressed with HER2, Luminal B as HER2+, Luminal B as HER2−, and Luminal A. This heterogeneity, in turn, generates differences in clinical behavior, pathological characteristics, and treatment responses, among others^5–7^.

An essential issue for treatment is the selection of the correct therapeutic modality, which largely depends on the Subtype of Breast Cancer (SBC)^8^. This is crucial because when BC is diagnosed early and managed with a multidisciplinary approach, it becomes a potentially curable disease^9^. To date, the estrogen receptor (ER), the progesterone receptor (PR), and the human epidermal growth factor receptor 2 (HER2) are utilized as biomarkers to streamline clinical decision-making, providing prognostic information, and predict responses to targeted therapies^10^. Currently, *BRCA1*, *BRCA2*, and *TP53* genes; STAT3, ESR1, and MUC-1 proteins; miRNAs (26b-5p, 124-3p, and 201-5p); and lncRNAs (HOTAIR, Linc-ROR, TROJAN), have been described as possible targets for treatment or diagnosis for BC^11–17^.

In this regard, several works aimed at identifying groups of genes, proteins, or consensus regions using different bioinformatics tools in diseases such as asthma, kidney, and colorectal cancer have been described in BC^18–20^. Therefore, identifying molecular targets can lead to a more accurate diagnosis of the type of BC, precisely targeted treatments, and potentially treatments that are less toxic than the current ones^21^. In this regard, it is possible to highlight some proteins as potential molecular targets, like Transmembrane Protein 170B (TMEM170B)^22^, Lipocalin 2 (LCN2)^23^, Macrophage Migration Inhibitory Factor (MIF)^24^, Signal Transducer and Activator of Transcription 3 (STAT3)^25^, Tumor Protein P53^26^, Vascular Endothelial Growth Factor A (VEGFA)^27^, Estrogen Receptor 1(ESR1)^28^, BCL2 Associated X (BAX)^29^, Ribonucleotide Reductase Regulatory Subunit M2 (RRM2)^30^, Matrix Metallopeptidase 1 (MMP1)^31^, and Maternal Embryonic Leucine Zipper Kinase (MELK), among others^32^. However, some of these molecules are biased to certain types of BC for a specific signal or were identified from a small biological sample^33^.

Nevertheless, it is essential to identify biomarkers according to each SBC to detect with high precision intra-tumor and inter-tumor heterogeneity in patients^34^, prognostic biomarkers^35,36^ that allow for understanding the degree of disease progression, as well as identifying possible specific therapeutic targets for each cancer subtype.

The amount of information on platforms such as The Cancer Genome Atlas (TCGA), International Cancer Genome Consortium (ICGC), or cBioPortal for Cancer Genomics makes information on gene and protein expression from cell lines, as well as tissue samples from different diseases, including breast cancer, increasingly accessible. Therefore, computational approaches are required to analyze the large amount of data^37^, such as the Weighted Gene Coexpression Network Analysis (WGCNA). This algorithm allows the identification of genes with similar coexpression profiles from different experiments or samples. Indeed, this algorithm has been implemented to identify potential biomarkers for diagnosis, prognosis, and therapeutic targets in diseases such as asthma, coronary artery disease, and different cancer types, like BC^18,38–41^.

Therefore, understanding the heterogeneity of BC from the gene expression profiles of cell lines and being able to identify genes expressed specifically for each of the main SBC would allow us to identify potential biomarkers that would improve our ability to diagnose this disease with greater precision, to understand its degree of progression, and even to identify specific therapeutic targets for each SBC that would enable us to determine the most appropriate treatments for patients^42^.

In this work, we identified 5 modules of genes with similar co-expression patterns and hub genes significantly associated with each of the Basal A, Basal B, Luminal A, Luminal B, and HER2 ampl SBC. By using the transcriptomic profiles of 49 breast cancer cell lines, through WGCNA and other bioinformatic methods. Our results provide novel possible biomarkers for specific SBC, which need to be explored for their potential.

## Materials and methods

### Datasets

**Figure 1:**
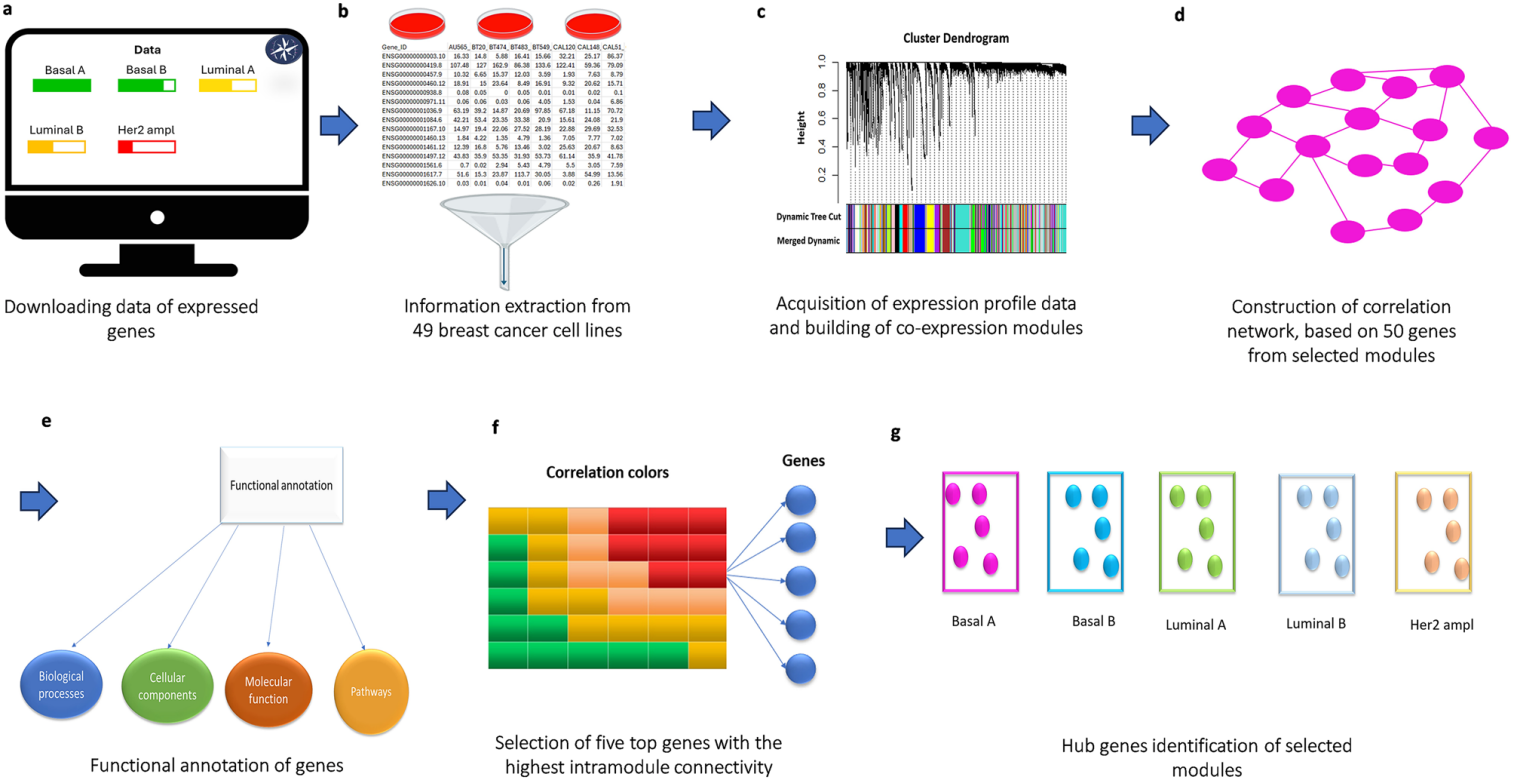
Workflow diagram outlining the proposed method for identifying modules and key genes associated with breast cancer subtypes using gene co-expression networks. (**a**) Initially, gene expression data from breast cancer cell lines were retrieved from the DepMap database version 21Q1. (**b**) A comprehensive analysis was then conducted on 49 breast cancer cell lines to obtain specific details about gene expression in these cell lines. (**c**) Next, a gene co-expression network was reconstructed based on the processed expression profiles using the WGCNA package in the R programming environment, resulting in 65 modules. (**d**) Subsequently, the module with the highest correlation for each breast cancer subtype (pink, turquoise, yellowgreen, skyblue, and navajowhite2) was selected to construct a correlation network consisting of 50 highly correlated genes from each chosen module. The software used for this process was Cytoscape. (**e**) Gene Ontology (GO) and Kyoto Encyclopedia of Genes and Genomes (KEGG)^48–50^ were then utilized to perform functional analyses aiming to understand the biological functions and metabolic pathways associated with the genes in the module. (**f**) The top five genes with the highest intramodule connectivity were identified. (**g**) Finally, the hub genes within each module were identified for each subtype of breast cancer.

The workflow chart of data preparation, processing, and analysis is illustrated in Fig. 1. The dataset used in this study (CCLE_expression) was downloaded from the DepMap, database version 21Q1 (https://depmap.org). This database contains gene expression data (RNAseq) for 1,376 cell lines (RSEM, gene) of 57,829 genes^43^, of which 49 cell lines related to BC were selected (Supplementary Table S1). Then, the data were normalized by using log2(TPM + 1) and filtered using the varFilter function in R, to exclude genes that exhibit less than 50% variation among samples, leaving a total of 28,143 expressed genes with greater variability for the subsequent steps.

Cell lines were classified according to the following features: by origin, 23 primary and 26 metastatic; and by subtypes: 15 Basal A, 8 Basal B, 12 Luminal A, 1 Luminal B, and 13 HER2 ampl (Supplementary Table S1)^4,44^.

### Weighted analysis of gene co-expression network

**Figure 2:**
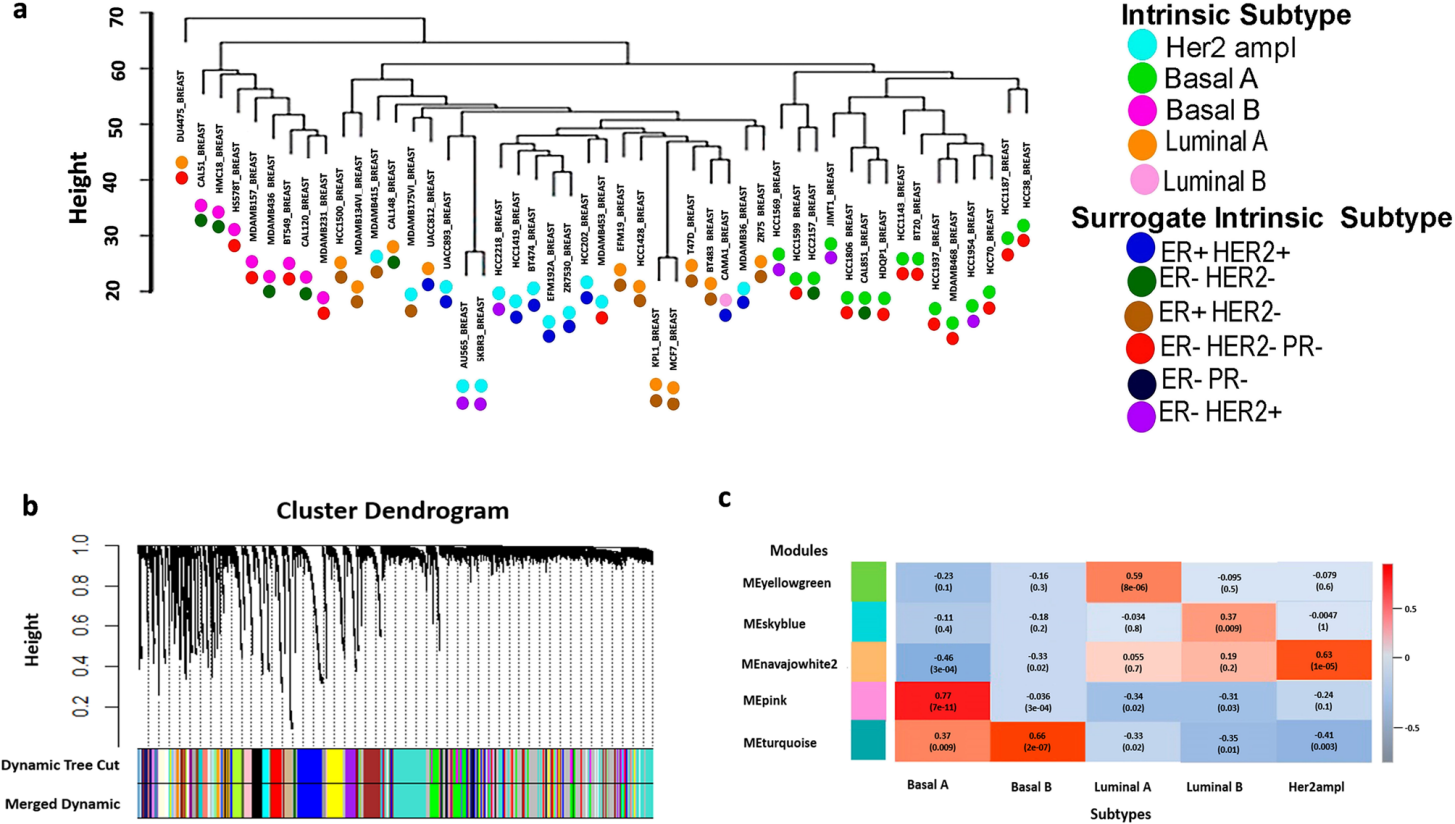
Weighted gene co-expression network analysis. (**a**) Clustering analysis to remove outliers**.** Sample dendrogram and trait indicator. Each color circle represents an SBC, five intrinsic subtypes, and six surrogate intrinsic subtype. (**b**) Network analysis of gene expression in BC identifying distinct modules of co-expression genes. (**c**) Correlations between module eigengenes and different SBCs with only the most significant module. The most correlated modules are shown for each SBC.

WGCNA^45^ was performed to evaluate the expression profile of the 49 cell lines and 28,143 genes of BC. To this end, a cluster analysis was conducted to identify atypical samples using the flashclust package in the R environment (Fig. 2a). Then, the biological network's scale-free topology property was incorporated by calculating the β parameter using SoftThreshold with a value of 14. An adjacency matrix was calculated using signal correlation networks and the Pearson correlation coefficient among all the genes. This adjacency matrix was transformed into a Topological Overlap Matrix (TOM), where a higher TOM value facilitates the identification of gene modules for each gene pair with strong interconnectivity. Afterwards, gene modules with similar expression patterns were identified using the average linkage hierarchical clustering algorithm, with default parameters (deepSplit set to 3 and a minimum module size set to 30). Additionally, modules with highly correlated eigengenes were merged based on a minimum height of 0.25 (mergeCutHeight function)^46^. Each module was identified with a different color, with the gray color reserved for uncorrelated genes^47^.

Enrichment analysis of all the modules that make up the network was performed to identify and interpret the biological functions based on the Kyoto Encyclopedia of Genes and Genomes (KEGG) database^48–50^ and Gene Ontology (GO), using the Annotation Visualization and Integrated Discovery (DAVID). All analyses were performed under the criteria of an adjusted *p* value of < 0.05.

To determine the association between each SBC and the modules, the module-trait relationship (MTR) was employed^46,47^. This approach was used to correlate the gene traits with the sample traits in each SBC, focusing on the most significant correlations.

### Identification of hub genes

In this study, we selected the top 50 genes with highly correlated values across different thresholds from each SBC in the modules, as detailed in Supplementary Table S2. This selection was made through the module of high module membership (MM), high genetic significance (GS), and high intramodular connectivity (IC). Afterward, we identified the five hub genes from each subtype, characterized by the highest intramodule connectivity, which is calculated as the sum of the degree of correlations between that gene and other genes within the module.

### Functional annotation

To evaluate the significance of the biological function of genes in each SBC, the Database for Annotation, Visualization, and Integrated Discovery (DAVID; version 6.8; https://david.ncifcrf.gov) was used. For this purpose, an enrichment analysis of Gene Ontology terms and KEGG pathways was implemented and displayed using the ggplot2 package in R, including molecular functions (MF), biological processes (BP), and cellular components (CC) with a statistical significance at an adjusted *p* value of < 0.05. Protein–protein interaction (PPI) networks were built using STRING software (version 10.5; http://string-db.org), with the interaction parameter set to confidence maximum of 0.90. Finally, to visualize and analyze the PPI network and the correlation network, Cytoscape software (version 3.6.1; http://www.cytoscape.org) was used.

### Validation of hub genes in an external dataset

To confirm hub shared genes in each SBC, the mRNA expression dataset of breast cancer was searched using the keywords: “breast cancer samples”, “TNBC”, “Luminal A”, “Luminal B”, “Her2”, “*Homo sapiens*”, “expression profiling by array” against the Gene Expression Omnibus (GEO) database (http://www.ncbi.nlm.nih.gov/geo). After a review, the GSE65194 (Affymetrix Human Genome U133 Plus 2.0 Array), GSE96860, GSE52194, and GSE134359 profiles, were selected. We conducted differentially expressed genes (DEG) analysis on the dataset using the R package “limma”. An adjusted *p* value < 0.05 was considered as the cutoff value, where our main hub genes and long non-coding RNAs were found for each SBC.

## Results and discussion

### Weighted analysis of gene co-expression network

To evaluate the gene expression of BC, RNA seq data from 49 cell lines comprising 28,143 genes were selected (Fig. 2a; Supplementary Table S3). These samples were used to construct a Gene Co-expression Network using the WGCNA software, with a Soft Threshold power β of 14 as the scale-free topology criterion, a signed network that allows for the identifying modules with more significant enrichment of functional groups, and Pearson correlation (Supplementary Fig. 1a,b)^45^. After, the network was processed by hierarchical clustering, identifying 65 modules (Fig. 2b); that is, a set of genes with similar expression patterns. Then, the eigengene modules were clustered to improve the reliability of the module divisions (Supplementary Fig. S2). The clustering algorithm was used to identify modules of highly correlated genes and was refined to improve the reliability of the module divisions, where each module was assigned a color, with the smallest containing 34 genes (yellow4) and the largest one containing 2297 genes (turquoise).

The modules were found to include genes previously associated with BC, such as *TP53*, which plays a key role in controlling cell division and cell death; *KI67*, related to cellular proliferation; *BRCA1*, *BRCA2,* which are hallmarks for hereditary BC^32^; and specific targets that coincide with the different types of BC, such as *BCL2L14,* which is apoptosis facilitator^51^, the tumor necrosis factor receptor superfamily member 1A (*TNFRSF1A)*^25^, and a potassium inwardly-rectifying channel subfamily J member 3 (*KCNJ3)*^52^, among others. Therefore, the role of these genes has already been demonstrated in BC, which supports the representativeness of the genes in our sample.

### Significant modules associated to SBCs

**Figure 3:**
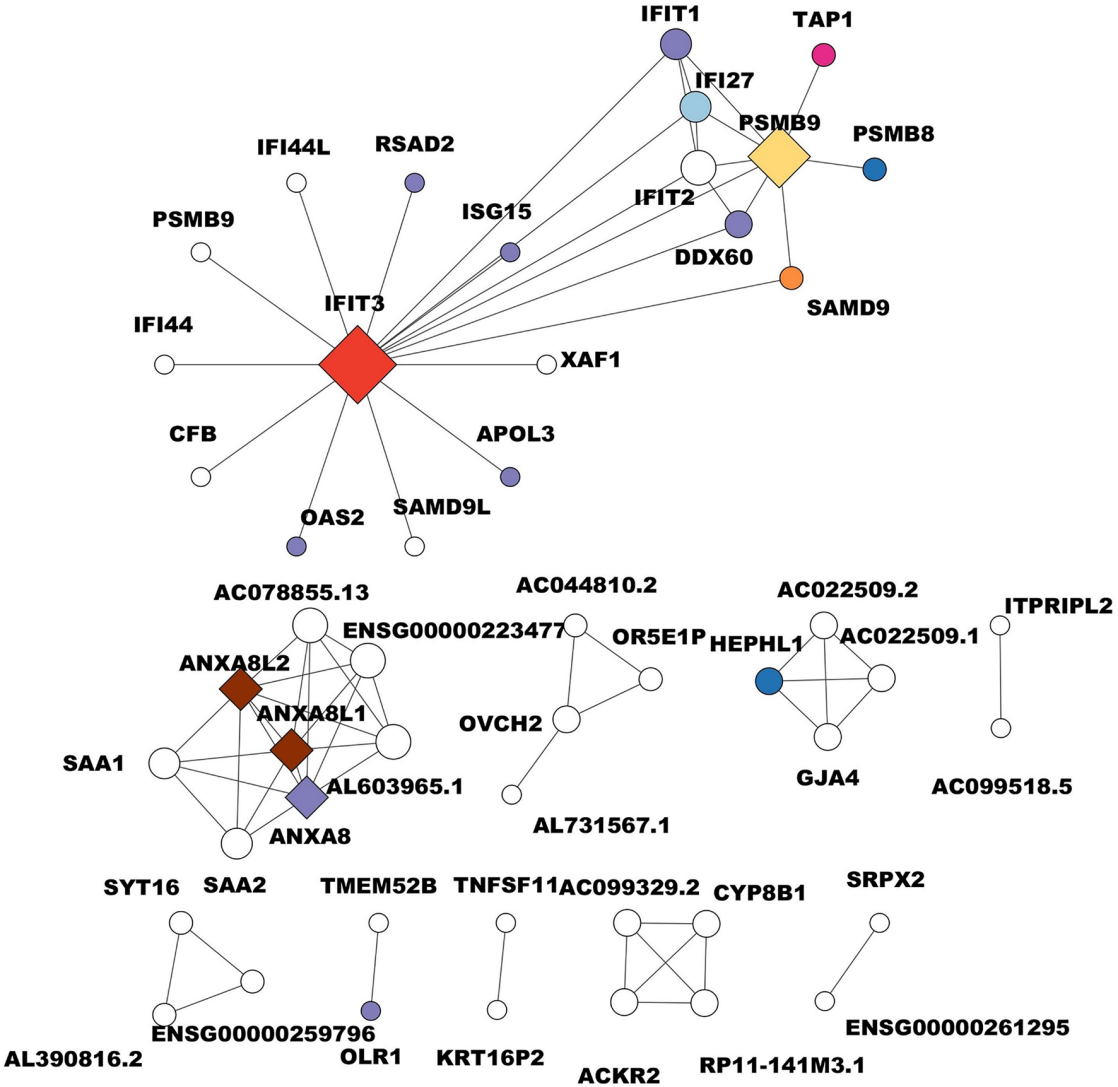
Basal A breast cancer subtype correlation network. The circles in the network represent different biological functions, each color-coded for easy identification. Dark blue circles represent catalytic activity, pink circles represent ATP-dependent activity, and brown circles represent calcium-binding proteins. Purple circles indicate binding functions, while yellow circles are involved in antigen processing to generate class I binding peptides. Red circles represent defense or immunity proteins, light blue circles represent signaling functions, and green circles represent transported activity. Lastly, orange circles represent the regulation of cell proliferation and apoptosis. The diamond shapes in the network represent hub genes, which are genes that have a high degree of connectivity in the network and play a crucial role in the biological processes. The size of each node (circle or diamond) corresponds to the number of connections or the degree of involvement in biological processes. Larger nodes have more connections, indicating a higher degree of involvement in various biological processes.

A Pearson correlation analysis was conducted to identify the most significant modules associated with each SBC. Below, we describe the most significant modules (Fig. 3, Supplementary Fig. S3). For Basal A, the pink module, with 879 genes (r = 0.77, *p* value = 7e−11) was found to be as significant. For Basal B, the turquoise module was identified with 2297 genes (r = 0.66, *p* = 2e−7); in the Luminal A subtype, the yellowgreen module, consisting of 131 genes (r = 0.59, *p* = 8e−6), was noted. For Luminal B, the skyblue module includes 169 genes with (r = 0.39 and *p* = 0.009). Finally, the navajowhite2 module representing HER2 ampl with 70 genes, was identified, showing r = 0.63 and *p* = 1e−5.

To associate the biological processes with the pink, turquoise, yellowgreen, skyblue, and navajowhite2 modules, which exhibit high correlation values, a Functional Enrichment of Gene Ontology Analysis with DAVID was achieved. In this regard, for Basal A, we identified 171 genes mainly involved in defense response (GO:0006952) with an adjusted *p* value of 2.60e−33 (Supplementary Table S3), regulating the body’s immune response, and in the body’s ability to contend against cancer cells which play a critical role throughout the course of breast carcinogenesis^53,54^. In breast cancer, specifically in the Basal A subtype, it has been shown that the immune system plays a dual role in tumor initiation and progression. This system can both inhibit and promote tumor expansion. Cytokinesas TGFβ, INFγ, and TNFα have been involved in both functions, by inducing different expression profiles in tumor cells^55^. Additionally, immune-related proteins that are overexpressed in this subtype of breast cancer have been associated with invasion and metastasis, as is the case with CD58^56^. Furthermore, the differences in the mutation profiles among subtypes underscore the complexity of this disease and the need for specific therapeutic approaches. In the Basal A subtype of breast cancer, a predominance of mutations in the *TP53* gene has been observed in 80% of cases. This high frequency is associated with increased invasion, migration, and cellular resistance, thereby contributing to tumor aggressiveness and a poor prognosis. This pattern suggests that alterations in *TP53* play a fundamental role in the pathogenesis of this subtype^57^. For Basal B, we found 370 genes involved in the movement of cell or subcellular components (GO:0006928) with an adjusted *p* value of 1.70e−24 (Supplementary Table S3). This movement is essential for many body functions, including tissue growth and repair, migration of immune cells to sites of inflammation, and cancer cells' invasion into surrounding tissues^58^. This is also consistent with work that has documented that patients with the Basal B subtype of breast cancer have a poor prognosis and earlier relapses with metastases. Moreover, metastases occur mainly in the brain, followed by the lungs and distal lymph nodes, suggesting that there are metastatic signatures for each organ^59^. Furthermore, significant overexpression of genes related to migration and invasion has been previously observed, such as *EGFR*, *MMP13*, *SOX4*, and *IGFBP2*^60,61^. Similarly, in the Basal B subtype, the prominence of mutations in *TP53* is maintained, followed by alterations *in PIK3CA*, *NF1,* and *FBXW7* in primary tumors. The mutations extend to ESR1, BRCA2, RB1, ERBB2, and AKT1 in metastatic cases, revealing a genetic complexity that affects treatment efficacy. This mutational diversity indicates the intrinsic heterogeneity of breast cancer tumors and the importance of identifying specific biomarkers to improve therapeutic approaches^62^.

For Luminal A, 16 genes were involved in the plasma membrane (GO:0044459) with an adjusted *p* value of 0.19 (Supplementary Table S4), regulating how cells attach to surrounding tissues and other blood vessels. These genes also control the migration and invasion of cancer cells, essential for tumor growth and cancer spread. They regulate how cells respond to cancer treatments, affecting the effectiveness of therapy^63^. Furthermore, the Luminal A subtype, as reported by Padua et al.^64^ observed that in the Luminal subtype, there was an effect on the expression of genes associated with the plasma membrane and the composition of the extracellular region, as well as the expression of the ERα receptor. This alteration may explain the characteristics of more aggressive cell growth. ERα is a crucial molecule in tumor development and progression due to its ability to activate various signaling pathways, whether cytoplasmic or nuclear, which are present, among other places, in the plasma membrane^65^. Additionally, this subtype shows low expression of genes related to cell proliferation^66^. On the other hand, in the Luminal A subtype, *PIK3CA* emerges as the most frequently mutated gene, followed by *GATA3*, *MAP3K1*, and TP53, reflecting a distinctive mutational profile that could influence therapeutic decisions^67^. This pattern suggests that*ESR1*, *GATA3*, *KMT2C*, and *PTEN* also play significant roles in cancer progression and treatment response^68,69^.

For Luminal B, 19 genes were associated with the extracellular space (GO:0005615) with an adjusted *p* value of 0.14 (Supplementary Table S4), which are important in cancer because they regulate how cancer cells interact with their immediate environment. These genes can control the production of proteins that modulate the adhesion of cancer cells to surrounding tissues and other blood vessels, essential for tumor growth and spread. In addition, these genes can also control the production of proteins that promote angiogenesis, and regulate how cells respond to cancer treatments, affecting the effectiveness of therapy^70^. The composition and three-dimensional structure of the ECM vary significantly, affecting processes such as apoptosis and cell proliferation. Furthermore, changes in the ECM have been linked to resistance to endocrine treatments and cancer recurrence, demonstrating the complexity of the interaction between tumor cells and their microenvironment^71^. Additionally, in the Luminal B subtype, a relationship has been established between the heterogeneity of breast cancer tumors and the components of the ECM, whose composition and three-dimensional structure undergo constant remodeling through closely regulated mechanisms during tumorigenesis. It has been evidenced that the ECM of different SBCs, such as Luminal and TNBC, exhibit disparities in the transcriptomic profile, especially in genes that influence cell behavior, such as apoptosis, the cell cycle, DNA replication, and the estrogen signaling pathway, including E-cadherin, vimentin, and ERα. Similarly, a correlation has been established between changes in ECM composition, endocrine resistance, and relapses^72^.

These subtypes also present a high expression rate of genes associated with proliferation, such as *MKI67* and *AURKA*^73^. The Luminal B subtype is characterized by a high frequency of mutations in *PIK3CA*, *GATA3*, and *TP53*, with a marker difference in the prevalence of P53 mutations compared to Luminal A^62^. This subtype also illustrates how the breast cancer genome can evolve during therapy, with 20% of patients acquiring mutations in *ESR1*, leading to resistance to endocrine therapy. This phenomenon underscores the importance of understanding mutational dynamics to address treatment resistance^74^. Finally, for HER2 ampl, four genes with coenzyme binding function were found (GO: 0050662) with an adjusted *p* value of 0.36 (Supplementary Table S5). These genes can control the production of enzymes involved in metabolic pathways, such as the pentose phosphate pathway, essential for the biosynthesis of molecules needed for cell growth and division^75^. In addition, Moasser et al.^76^ highlights the overexpression of the HER2 gene and its implication in an aggressive tumor phenotype through the activation of signaling pathways such as RAS/MAPK and PI3K/AKT. This subtype shows a clear association between the enzymatic function of involved proteins and a higher propensity for cell proliferation and survival of the HER2-positive subtype.

Approximately 20% of cases exhibit amplification or overexpression of HER2, associated with an aggressive tumor phenotype and poor survival. HER2 encodes for a transmembrane tyrosine kinase receptor, playing a crucial role in cell–cell and cell–stroma communication through signal transduction. Many proteins and intermediaries are involved in these pathways exhibiting enzymatic activity. The main signaling pathways involved, RAS/MAPK, PI3K/AKT, and JAK/STAT, among others, affect cell proliferation, survival, mobility, and adhesion^77^. In this subtype, gene enrichment is primarily related to coenzyme-binding function^76^. Furthermore, this subtype has the second-highest prevalence of P53 mutations (70%), making it a significant biomarker and therapeutic target^78^. Additionally, three mutations (Q429R, Q429H, and T798M) in HER2, identified in the MCF7 cell line, have been shown to diminish the effectiveness of trastuzumab in this cell line, and are also postulated as potential biomarkers for predicting resistance to therapy^79^. These results demonstrated that the SBCs, Basal A, Basal B, Luminal A, Luminal B, and HER2 ampl, exhibit different co-expression patterns, thereby implicating divergent biological processes. This distinction could facilitate the identification of potential biomarkers unique to each subtype.

In Basal A, for metabolic pathways analysis, 21 genes are involved in the phagosome pathway with an adjusted *p* value of 2.4e−5, and 23 genes are involved in Epstein–Barr virus infection with an adjusted *p* value of 8.0e−5. A meta-analysis indicates a strong statistical relationship between Epstein–Barr virus infection (EBV) and BC risk, suggesting a potential role for EBV infection in the development of BC^80^. Gupta et al.^81^ reported that the presence of EBV in triple-negative breast cancer poses a high risk for patients. Additionally, there is a potential association between serous epithelial ovarian cancer (EOC) and EBV infection, which could be subtype-specific^82^. For the Basal B subtype, 52 genes participate in proteoglycans in cancer with an adjusted *p* value of 3.1e−7 (Supplementary Table S6). Proteoglycans contribute to the development and progression of the disease and its response to treatment by potentially affecting tumor growth, invasion, and metastasis through interactions with other extracellular matrix molecules and the regulation of signaling pathways^83^. These results illustrate that the genes in the pink module, highly correlated with the Basal A subtype, differ from those in the turquoise module, highly correlated with the Basal B subtype, involving them in distinct biological processes and molecular functions.

### Correlation network and identification of hub genes

Hub genes are characterized by being genes whose degree value is higher than the average of the threshold network degree value^84^. Many studies have shown that the relationship between connectivity and node significance carries important biological information^47^. In this respect, to identify hub genes for each SBC, we obtained the top 50 highly degree genes for pink, turquoise, yellowgreen, skyblue, and navajowhite2 modules, and their networks were displayed (Supplementary Tables S7, S8). The results show that the Basal A network is composed of 50 nodes and 75 edges with a threshold of 0.05. The gene with the highest degree, corresponding to the main hub gene, is *IFIT3* (Interferon-induced protein with tetratricopeptide repeats 3) with 16 degrees, followed by *PSMB9* (Proteasome 20S Subunit Beta 9) with 12 degrees, *anxa8l2* (annexin A8-like 2), *ANXA8* (annexin A8), and *ANXA8L1* (annexin A8-like 1) with 7 degrees each one (Fig. 3, Supplementary Table S9). In this regard, *IFIT3* encodes for a transcription factor involved in the negative regulation of apoptotic processes and the negative regulation of cell proliferation^85,86^, indicating its potential role in cancer development and progression.

It inhibits proliferation in human BC cells (treated with T-47D curcumin)^87^, which is consistent with the biological process identified for the pink module. Moreover, *ifit3* is considered a predictive biomarker for chemotherapy and radiation in some human cancers, such as breast, lung, prostate, neck, head cancer, and glioma^86,87^, indicating its potential utility across multiple cancer types. However, this is the first time that it has been identified as a potential biomarker associated with the Basal A subtype. Recently, Lamsal et al.^88^ demonstrated that *IFIT3* mRNA and its protein are highly expressed in human metastatic breast cancer cell line MDA-MB-231, while its expression is low in non-metastatic MDA-MB-453 cell line. The next hub gene, *PSMB9*, encodes a modified enzyme with peptidase activity involved in the proteasome pathway, which helps cells deal with oxidative and proteotoxic stress. The overexpression of proteasomes is present in a wide variety of cancer types^89^, further highlighting the importance of these genes in cancer progression and treatment response. Proteasomes play a key role in the stress response because they degrade damaged proteins^90^. *PSMB9* is also a potential diagnostic biomarker and tumor suppressor for human uterine leiomyosarcoma^91^. ANXA8L2 Protein 2, ANXA8 protein (annexin A8), and ANXA8L1 protein 1 are members of the annexin family of evolutionarily conserved Ca^2^^+^ and phospholipid-binding proteins; their overexpression has been associated with acute myelocytic leukemia^32^. In addition, *ANXA8L1* participates in biological regulation, cellular process, localization, metabolic process, calcium-binding protein, response to wounding, and endomembrane system organization^92,93^. Interestingly, *ANXA8* expression is directly related to a subgroup of basal-type breast cancer patients with poor prognosis^94^, which correlates with the association identified between these three hub genes and the Basal A subtype in this work.

**Figure 4:**
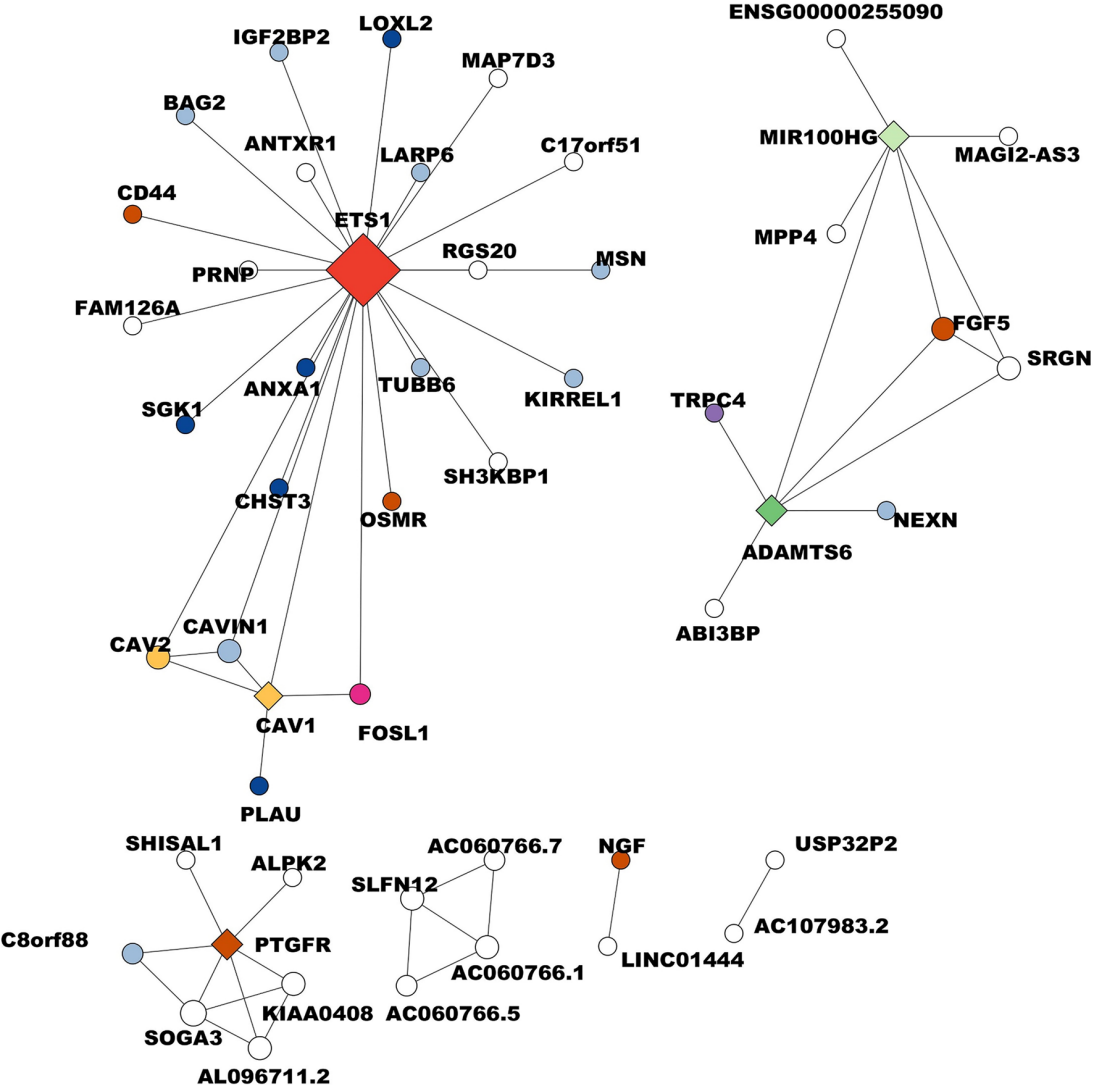
Basal B breast cancer subtype correlation network. The circles in the network symbolize different biological functions, each distinguished by a unique color. Dark blue circles represent catalytic activity, pink circles represent transcriptional regulator activity, and brown circles symbolize molecular transduction activity. Light blue circles indicate binding functions, while yellow circles are associated with molecular adaptor activity. Red circles represent gene-specific transcriptional regulators, purple circles indicate transporter activity, green circles represent modifying enzymes, and light green circles indicate regulators of cell proliferation. The diamond shapes in the network represent hub genes. These are genes that have a high degree of connectivity within the network and play a significant role in the biological processes. The size of each node, whether a circle or diamond, corresponds to the number of connection degrees involved in biological processes. Larger nodes have more connections, indicating a higher degree of involvement in various biological processes.

In the case of the Basal B subtype, the network consists of 50 nodes and 57 edges with a threshold of 0.0670. The main hub gene is the transcription factor *ets1* (23 degrees). The remaining four hub genes are *ADAMTS6* with 6 degrees, MIR100HG with 6 degrees, *PTGFR* with 6 degrees, and *CAV1* with 5 degrees (Fig. 4, and Supplementary Table S10). *ETS1* belongs to the ETS family of transcription factors and is overexpressed in some types of cancer cells, such as prostate cancer and leukemia^95^. It participates in cancer pathways like angiogenesis, PDGF, VEGF, and RAS signaling, cellular senescence, human T-cell leukemia virus infection, and renal cell carcinoma. The biological processes of *ETS1* include extracellular structure organization, response to wounding, blood vessel development, negative regulation of cell proliferation, regulation of cell death, DNA-binding, and transcription regulator activity^92,93^.

It was detected as a potential prognostic biomarker in gastric cancer^96^. Recently, *ETS1* was also described as a specific prognostic biomarker for TNBC patients, but not for patients who are not triple-negative or other patients with breast cancer. In addition, a very close association with invasiveness, metastasis, and a poor prognosis was determined in TNBC patients, which is directly related to the overexpression it induces of the genes that code for the metalloproteinases MMP1, MMP-3, and MMP9, as well as VEGF among others^97^. This result agrees with the one obtained in this work where the hub *ETS1* gene is directly associated with the Basal B subtype. *ADAMTS6* has catalytic activity and is involved in cellular processes, protein-modifying enzymes, extracellular structure organization, and blood vessel development. It contains a disintegrin and metalloprotease domains with thrombospondins motifs, members of this family can be secreted by cancer and stromal cells and can contribute to modifying the tumor microenvironment by multiple mechanisms, such as cell adhesion, migration, proliferation, and angiogenesis^98^. Additionally, *ADAMTS6* suppresses tumor progression via the ERK signaling pathway and is a prognostic marker in human BC^99^.

The next hub gen is MIR100HG, a long non-coding RNA (lncRNAs). It acts as a regulator of cell proliferation and has been reported as a prognostic biomarker for gastric cancer^100^. LncRNAs are endogenous in various cancers, including BC^101^, and are involved in different biological processes like migration, metastasis, and proliferation^17^. *PTGFR* is a tumor suppressor gene involved in a tumor promotion process, perhaps via interaction with its specific ligand (PGF2α). It has been described as a potential biomarker to predict prostate cancer progression, particularly between stage II and subsequent stages of the disease^102^. *CAV1* is a membrane protein with a crucial role in the advancement of BC, including cell proliferation, apoptosis, autophagy, invasion, migration, and metastasis^103^. *CAV1* has been identified as a biomarker of colorectal, lung, penile, prostate, and non-small cell lung cancer^104–108^. Interestingly Al et al. found genes identified from samples of patients with BC of basal subtype to *IFI44L* (Basal A), *PLAU*, and *IL6* (Basal B), which are in the top 50 that we identified of these subtypes^109^.

**Figure 5:**
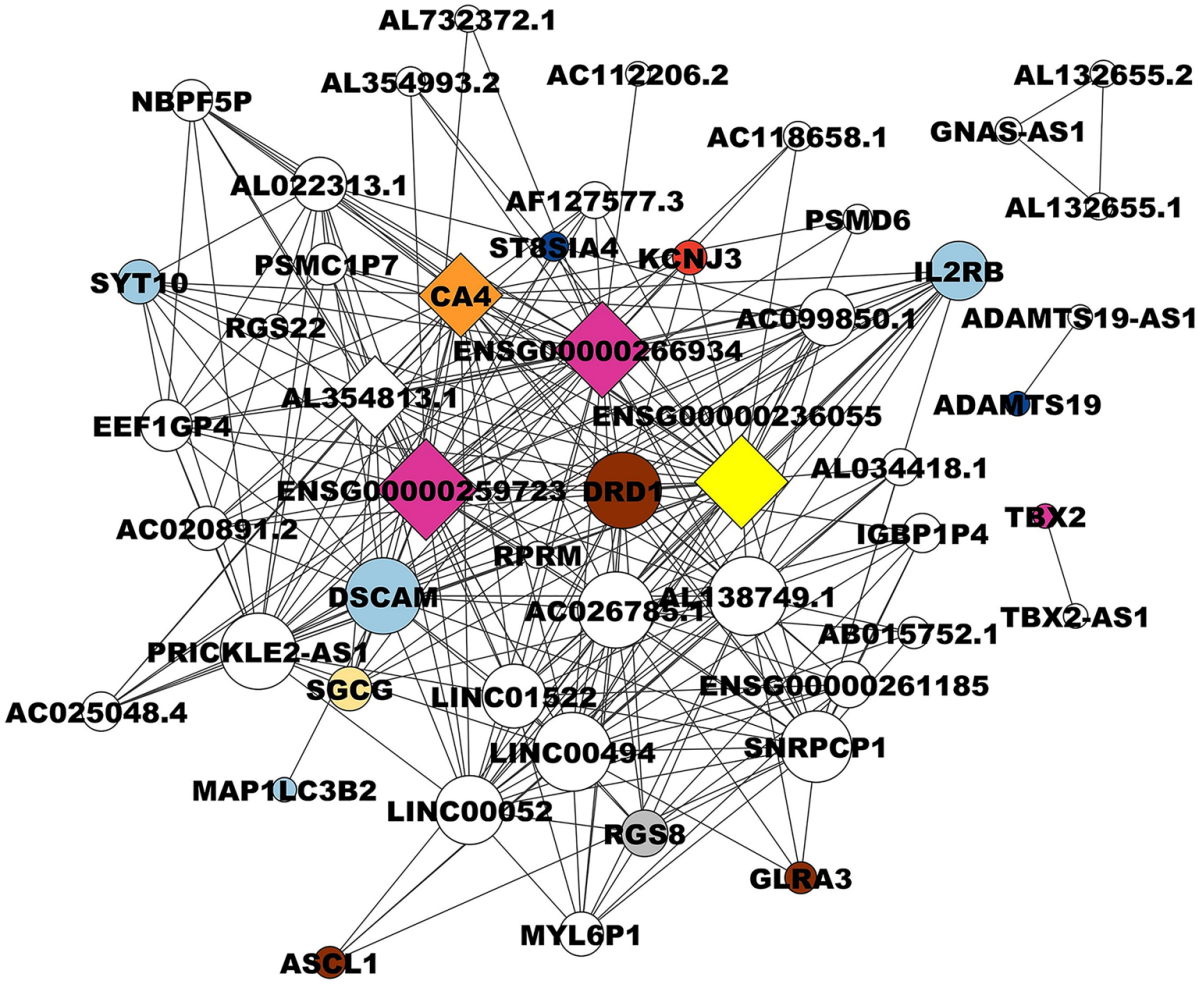
Representation of a correlation network for the Luminal A subtype of breast cancer. Visualising the relationships between different biological functions and hub genes. The circles in the network represent different biological functions, each distinguished by a unique color. Dark blue circles denote catalytic activity, pink circles represent transcriptional regulator activity, and brown circles symbolize molecular transduction activity. Light blue circles indicate binding functions, while orange circles are associated with metabolite interconversion enzyme activity. Red circles represent transporter activity, purple circles indicate transmembrane signal receptor activity, yellow circles indicate cytoskeletal protein functions, and gray circles represent protein-binding activity modulators. The diamond shapes in the network represent hub genes, which are genes with a high degree of connectivity within the network, indicating their important role in the biological processes. The size of each node, whether a circle or diamond, corresponds to the number of connection degrees involved in biological processes. Larger nodes have more connections, indicating a higher degree of involvement in various biological processes.

For subtype Luminal A, we identified a network with 50 nodes and 264 edges with a threshold of 0.0445. The gene with the highest degree and the main hub gene is the transcriptional regulator ENSG00000259723 with 32 degrees, followed by ENSG00000266934 with 30 degrees, ENSG00000236055 with 29 degrees, *CA4* with 25 degrees, and AL354813.1 with 24 degrees (Fig. 5, Supplementary Table S11). ENSG00000259723 is a lncRNA expressed in seven cancers, such as bladder cancer, colon adenocarcinoma, head and neck squamous cell carcinoma, lung squamous cell carcinoma, ovarian cancer, prostate adenocarcinoma, and endometrial carcinoma of the uterine body^110,111^. ENSG00000266934 is present in 31 species, such as *Gorilla gorilla gorilla*, *Carlito syrichta*, *Pteropus vampyrus*, *Dasypus novemcinctus*^112^; and ENSG00000236055, located on chromosome 17, and 1 respectively. *CA4* is a carbonic anhydrase implicated in cell proliferation, and inhibits cell proliferation, invasion, and metastasis^32^. *CA4* has been reported as a clinicopathological biomarker of immune infiltration and outcomes in kidney carcinoma, lower-grade glioma, pulmonary adenocarcinoma, and uveal melanoma^113^, and identified as a biomarker for gastric cancer^114^. AL354813.1 is a lncRNA located on chromosome 20 and has one transcript with 2437 bp^110^. Recent research has shown that the amount of IncRNA in cancer differs in normal tissues, and there are many different prognostic-related expressions. In other types of cancer, lncRNAs have been identified as prognostic markers^115,116^. It was found that lncROPM plays a crucial role in sustaining and has functioned as a chemo-resistance biomarker in BC cancer stem cells^117^. Although many lncRNAs have been identified in BC, their functional mechanism remains unknown. Interestingly, we found not only hub genes that encode transcription factors but also lncRNAs, as has also been reported^118,119^.

**Figure 6:**
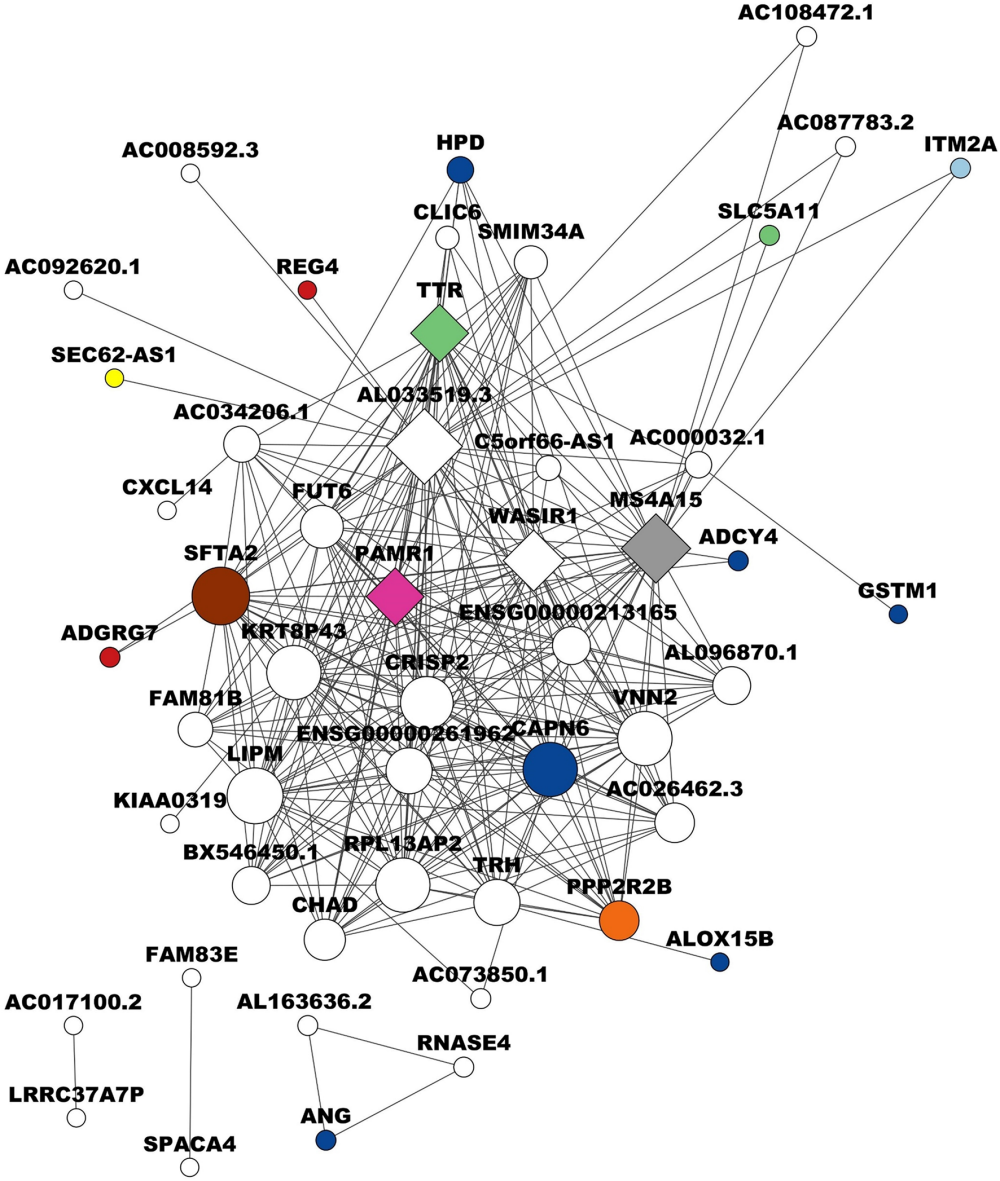
Correlation network for the Luminal B subtype of breast cancer. The circles in the network symbolize different biological functions, each distinguished by a unique color. Dark blue circles denote catalytic activity, pink circles represent proliferation inhibitor activity, and brown circles symbolize disease-susceptibility for panbronchiolitis. Light blue circles indicate binding functions, while orange circles are associated with molecular function regulator activity. Red circles represent molecular transducer activity, green circles denote transporter activity, and gray circles represent signal transduction functions. The diamond shapes in the network represent hub genes. These are genes that have a high degree of connectivity within the network, indicating their significant role in the biological processes. The size of each node, whether a circle or diamond, corresponds to the number of connection degrees involved in biological processes. Larger nodes have more connections, indicating a higher degree of involvement in various biological processes.

The network for the subtype luminal B is composed of 50 nodes and 265 edges, with a threshold of 0.043. The gene with the highest degree is *AL033519.3* with 36 degrees, *MS4A15* with 32 degrees with signal transduction activity, *WASIR1* with 27 degrees, and *PAMR1* with 25 degrees, and *TTR* with 25 degrees (Fig. 6, Supplementary Table S12). *AL033519*.3 is a hypothetical gene located on chromosome 6 and comprises 771 nucleotides. Currently, there are s no studies linking these genes to tumorigenesis or its progression. *MS4A15* is involved in cellular processes, response to stimulus, and signaling^32^. It regulates the cellular metabolism involved in the malignant transformation of numerous tumors, including ovarian cancer^120^. Its overexpression correlates with poor survival in patients with ovarian cancer, making it a promising therapeutic target. *MS4A15* has also been suggested as a possible biomarker for colon and gastric cancer^121^. *WASIR1* is a lncRNA that appears to interact with *LINC00511,* which is overexpressed in BC and promotes its progression^122^. *PAMR1* is predicted to be involved in proteolysis^32^. It is inactivated by promoter hypermethylation in BC, therefore, it has been considered a tumor suppressor^123^ and a potential biomarker that has a negative correlation to the spread of cervical cancer^124^. *TTR* plays a role in stimulating tumor growth mediated by activation of mitogenic and oncogenic molecules, as well as immune and endothelial cells, specifically in lung cancer. It has a significant effect on the range of functions of endothelial cells, managing both tumor and immune cell migration and infiltration. When endothelial cells are treated with *TTR*, there’s a suppression of T cell proliferation^125^ linking it to disease pathologies and marking it as Oxidative Stress Biomarker^126^. Additionally, the inhibition of *TTR*, associated with conditions such as malnutrition and inflammation, is recognized as a biomarker for various human morbidities^127^.

**Figure 7:**
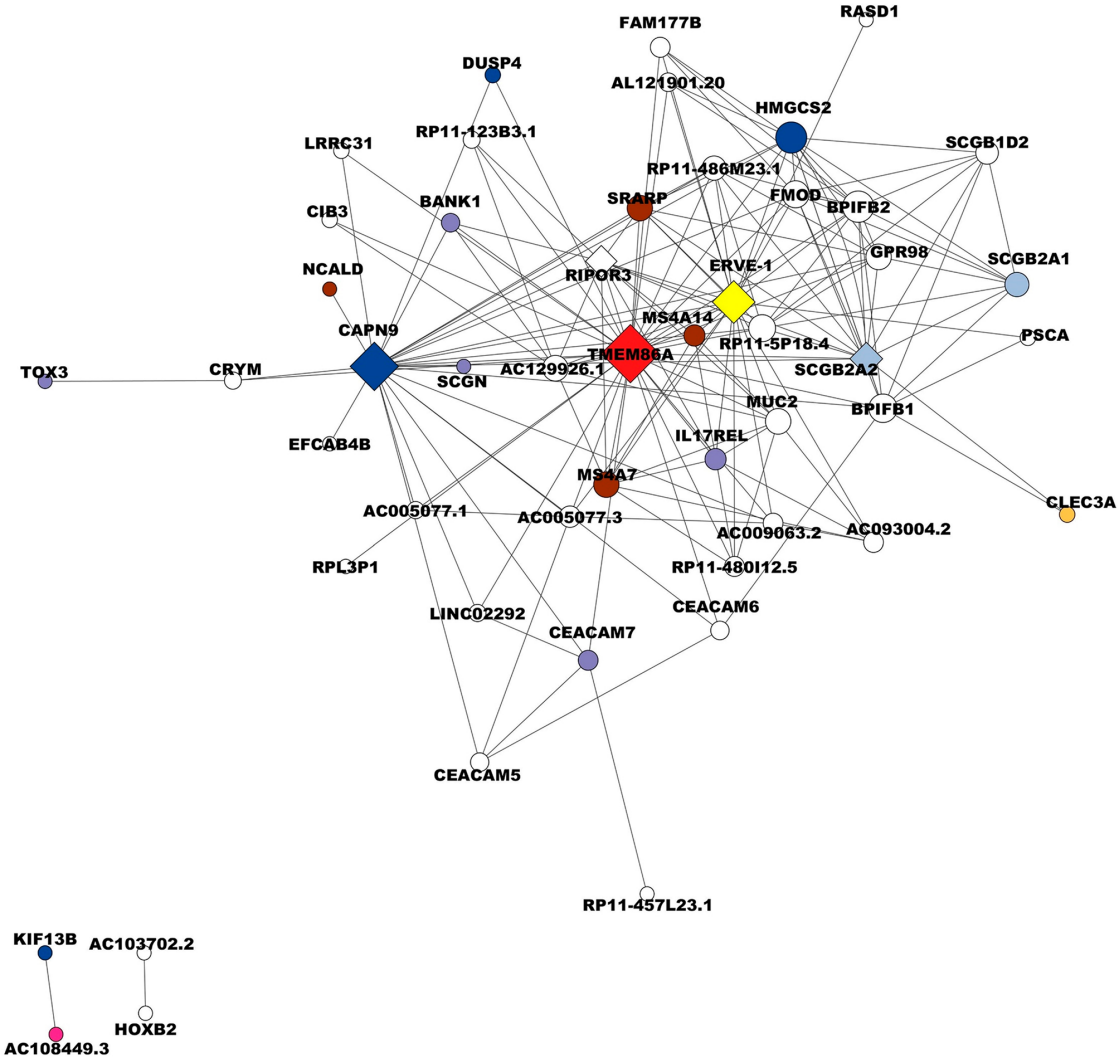
Visualisation of a correlation network for the HER2 ampl subtype of breast cancer. The circles in the network represent different biological functions, each distinguished by a unique color. Dark blue circles denote catalytic activity, pink circles represent ATP-dependent activity, and brown circles symbolize biological regulation. Purple circles indicate binding functions, while yellow circles are associated with multicellular organismal processes. Red circles represent functions linked to autosomal recessive congenital ichthyosis, and light blue circles denote signaling functions. The diamond shapes in the network represent hub genes, which have a high degree of connectivity within the network, indicating their important role in biological processes. The size of each node, whether a circle or diamond, corresponds to the number of connection degrees involved in biological processes. Larger nodes have more connections, indicating a higher degree of involvement in various biological processes. This image is a valuable tool for visualizing complex biological interactions and understanding the role of different functions and genes in HER2 ampl.

Finally, the network for the subtype HER2 ampl consists of 50 nodes and 162 edges with a threshold of 0.0211. The gene with the highest grade and considered as the main hub is *TMEM86A* with 32 degrees, linked to ichthyosis disease; followed by four important genes in this network which are *CAPN9* with 25 degrees, *ERVE*-1 with 21 degrees, *SCGB2A2* with 14 degrees, and *RIPOR3* with 13 degrees (Fig. 7, Supplementary Table S13). *TMEM86A* is a lysoplasmalogenase that may be important for regulating keratinocyte membrane properties in terminal differentiation cells^128^ proposed as a candidate for tumor surface antigen in HER2 ampl BC, which coincides with our results^129^. *CAPN9*, the calpains are intracellular cysteine proteases that function in various important cellular processes, including signaling, motility, apoptosis, and cellular survival^130^. Down-regulated *CAPN9* is related to poorer patient clinical outcomes following endocrine therapy in BC^131^. *ERVE-1* is an endogenous retroviral sequence from the family ERVs (endogenous retroviral sequence) detected in several cancers, while they remain silent in healthy tissues^132^. However, in BC, the expression of *HERV*-K (human endogenous retrovirus type K) is frequent, and HERV-K (HML-2) antibodies and mRNA tended to be higher in BC patients with a primary tumor, who later will develop the metastatic disease than in patients who did not develop cancer metastasis^133^. It is important to note that most of the HER2 ampl cell lines that used for this analysis are metastatic. *SCGB2A2* encodes a glycoprotein indicated as a potential diagnostic and prognostic marker for BC^134^. It is involved in biological regulation, cellular processes, response to stimulus, and signaling. A genetic rearrangement has been detected in breast tumors that overexpress *SCGB2A2,* suggesting changes in transcriptional regulation as a cause of overexpression^135^. Its expression does not seem to be affected by steroid hormones^135,136^. *SCGB2A2* is a biomarker for the early detection of bone marrow micrometastasis^134^. A downregulation of *RIPOR3* in tongue cancer is associated with a worse prognosis. Its expression is related to the modulation of immune pathway function and hypermethylation, making it a promising prognostic biomarker and is linked to the immune cell infiltration of cell carcinoma of the mobile tongue^137^. The functions and roles of the hub genes described above for each SBC underscore their significance in breast cancer or other cancer types. Our results demonstrate that each SBC evaluated (Basal A, Basal B, Luminal A, Luminal B, and Her2 ampl) presents a different gene expression pattern, identifying highly correlated hub genes for each subtype. Moreover, some of these genes are involved in similar biological and cellular processes including proliferation, invasion, metastasis, and immune response, among others. Therefore, these hub genes may be potential diagnostic biomarkers for each SBC.

### Protein–protein interaction (PPI)-networks for each SBC

The interactions between the proteins encoded by the hub genes of each module can provide insights into the pathways or mechanisms activated in the different SBCs. For this reason, we analyzed the PPIs in the STRING database. The results showed that the PPI-network for Basal A consists of 33 nodes and 93 edges (Supplementary Fig. 4a). The protein most closely connected is ISG15 ubiquitin-like protein, which functions as a cytokine to modulate immune responses such as viral infections (14 degrees). Interestingly, its expression has been relevant in the pathogenesis of BC due to its role in motility, invasion, metastasis, and a poor response to chemotherapy and radiotherapy^138,139^. Additionally, XAF1 counteracts the inhibitory effect of apoptosis (13 degrees)^140^, IFIT2 involve positive regulation of the apoptotic process and response to the virus (13 degrees)^141^, RSAD2 is an interferon-inducible antiviral protein (13 degrees), and OAS2 involved in the innate immune response to viral infection (13 degrees)^142^. The Basal B network consists of 40 nodes and 28 edges. The most connected protein is CAV1, recognized as a tumor suppressor (10 degrees) with a dual role in BC progression^103^; CD44, which participates in cell adhesion and tumor metastasis, among others (9 degrees)^143^ PLAU related to apoptotic pathways in synovial fibroblast (4 degrees)^144^; PTRF, plays a significant role in caveolae formation, ribosomal transcriptional activity in response to metabolic challenges in the adipocytes, among others (3 degrees); CAV2 involve in signal transduction, lipid metabolism, cellular growth control, and apoptosis (3 degrees)^32^ (Supplementary Fig. 4b). The PPI network for Luminal A consists of 17 genes and 0 edges (Supplementary Fig. 4c). The Luminal B network includes 29 genes and four edges, with the most connected protein being ANG, which induces angiogenesis (2 degrees)^145^; and TRH releasing mature thyrotropin, a hypothalamic regulatory hormone (2 degrees)^32^; CAPN6 may play a role in tumor formation by inhibiting apoptosis and promoting angiogenesis (1 degree)^146^; TTR transports thyroid hormones and participates in proteolysis, nerve regeneration, autophagy, and glucose homeostasis (1 degree)^32,147^; ITM2A is a type II membrane protein that may be involved in osteo- and chondrogenic differentiation (1 degree)^148^ (Supplementary Fig. 4d). The PPI-network for HER2ampl consists of 34 genes and nine edges. The protein which the highest connection is SCGB2A2, involved in the androgen receptor signaling pathway (3 degrees)^149^; SCGB1D2 a chemotherapeutic agent widely used for prostate cancer (2 degrees)^32^; BPIFB1 involved in the innate immune response to bacterial exposure in the mouth, nasal cavities, and lungs (2 degrees); SCGB2A1 bind androgens and other steroids, a chemotherapeutic agent used for prostate cancer, under the transcriptional regulation of steroid hormones (2 degrees); CEACAM5 plays a role in cell adhesion, intracellular signaling, and tumor progression(2 degrees)^32,150^ (Supplementary Fig. 4e).

### Hub gene expression in BC patients

We conducted a DEG on the GSE65194, GSE96860, and GSE52194 datasets, revealing that our main hub genes, IFIT3, ETS1, CA4, and TMEM86A, were overexpressed in each SBC with their respective adjusted *p* values, while *MS4A15* only showed significant overexpression in dataset GSE52194 (Supplementary Table S14). Interestingly, *IFIT3* has been recognized as a possible biomarker for predicting the response to chemotherapy and radiation in specific human cancers (breast, lung, prostate, head and neck cancer, and glioma)^86,151^. Additionally, the expression of *IFIT3* mRNA could function as a predictive marker for the efficiency of immunostimulant treatments in breast cancer cells^152^. Recently, *ETS1* was identified as a predictive biomarker for TNBC, notably enhancing the progression of TNBC by activating the YAP signaling pathway^97^, aligning with our findings. *CA4* has been associated with the prognosis of lung adenocarcinoma^153^. Finally, *TMEM86A* has been suggested as a candidate for a tumor surface antigen in HER2 ampl BC^129^, and another for differential expression in various cancer types, including liver, colon, and gastric^154^. A differential gene expression analysis on the GSE134359 dataset, which includes long non-coding RNA (lncRNA) from SBCs, identified ENSG00000259723 and AL033519.3 as the main hub genes for Luminal A and Luminal B, with an adjusted *p* values of 3.43E−01 and 2.46E−05, respectively. In breast cancer, lncRNA functions as both a promoter and inhibitor of metastasis^115^. It is important to recognize that research into lncRNAs in breast cancer is still evolving, with more comprehensive studies required to fully grasp their functions and mechanisms. Nevertheless, progress in this area has already demonstrated that lncRNAs significantly impact BC biology. They offer promising potential as therapeutic targets, highlighting the importance of continuing exploration in this domain^21^.

Ultimately, the search for molecules capable of identifying and distinguishing SBCs, as well as serving as therapeutic targets, remains a critical endeavor in cancer treatment. Correct diagnosis enables the administration of the most effective treatments. Moreover, it is crucial to monitor treatment efficacy, prevent cancer cell resurgence, and avert the development of drug resistance, all to eliminate breast cancer cells. Our research underscores the identification of pivotal genes whose overexpression is specifically correlated with certain SBC. While further validation in diverse patient cohorts of various SBCs is necessary, our findings align with those of Charafe-Jauffret et al.^155^, who utilized 31 breast cancer cell lines and DNA microarrays to identify, among others, three key genes we also recognized: *ANXA8* (Basal A), *ETS1* and *CAV1* (Basal B), associating them with the basal subtype of breast cancer. Calderon-Gonzalez et al.^156^ and Stein et al.^157^ have also shown that *ANXA8* is overexpressed in basal subtype cell lines of both human and mouse origin. Regarding *ETS1*, its association with local metastasis in TNBC patient samples was noted^97^. Xie et al.^99^ reported *adamts6* (Basal B) as a potential prognostic marker in breast cancer, supported by a multivariate analysis of patient tumor samples and cell lines (Supplementary Table 15).

In the case of the Luminal A subtype, the *ca4* gene hub identified in our study has also been detected and proposed as a prognostic biomarker from samples of patients with invasive cancer^158^. For the Luminal B subtype, none of the gene hubs identified in this work have been proposed as potential biomarkers in other studies. However, Wu et al.^159^ suggest the *capn6* gene as a prognostic marker for BC. While it is not the main gene hub correlating with this SBC in our analysis, it is present in the skyblue module. Similarly, the presence of *PPP2R2B*, *HPD*, *ADGRG7*, *ADCY4*, and *ITM2A* supports the correlation of the expression of these genes with the Luminal B subtype (Supplementary Table 15), underscoring the complexity and the nuanced understanding required to navigate the molecular landscape of SBCs. Additionally, these insights highlight the potential of specific genes to serve as biomarkers for BC, emphasizing the need for further research to validate these associations and understand their implications for diagnosis and treatment strategies.

Finally, regarding the gene hubs associated with the HER2 ampl subtype^134^, *SCGB2A2* is predominantly overexpressed in BC patients and shows a high correlation with increased expression and the presence of micrometastases^134^. The similarities and differences in identifying these gene hubs may stem from the origin of the samples, whether they are cell lines or patient tumor samples. While these sources have been shown to possess great similarities^160^, they also exhibit certain differences, particularly when compared with samples from advanced-stage tumors^161,162^. These disparities may also relate to the number of samples used, the types of algorithms applied, and the specific correlation or approach sought in the research. Additionally, this variability underscores the well-documented heterogeneity in breast cancer, highlighting the complexities of diagnosing and treating this multifactorial disease.

## Conclusions

Identifying biomarkers that enable the precise diagnosis of specific SBC is crucial for understanding the type of tumor, its prognosis or stage, and selecting the most effective treatment. This study identified a specific module with the highest correlation for each SBC, pinpointing at least one hub gene with the highest correlation for each subtype: Basal A: *IFIT3*; Basal B: *ETS1*; Luminal A: ENSG00000259723; Luminal B: AL033519.3; HER2ampl: *TMEM86A*. These genes are involved in defense response, cell process regulation, and transport for each respective SBC.

Validation analysis of the highly connected hub genes expressed in patients with SBCs, using the external dataset GSE65194, revealed significant differential expression of *IFIT3*, *ETS1*, *CA4*, and *TMEM86A.* Our findings could provide a theoretical basis for enhancing prognosis and diagnosis through potential SBC biomarkers. Nevertheless, the mechanisms underlying these associations need to be explored in future studies.

This research proposes further analysis of these targets as potential biomarkers. It represents the first study to use a large sample of breast cancer cell lines and WGCNA to identify potential biomarkers associated with SBCs. The development of co-expression networks in each SBC may elucidate possible biomarkers for risk assessment, prognostic determination, screening, differential diagnosis, treatment response prediction, and disease progression monitoring. These hubs could be promising targets in drug development for monitoring or treating SBCs. A significant strength of this study is its focus on SBCs rather than BC in general, offering a more nuanced understanding of subtype-specific biomarkers.

### Supplementary Information

Supplementary Information.

Supplementary Figure 1.

Supplementary Figure 2.

Supplementary Figure 3.

Supplementary Figure 4.

Supplementary Table 1.

Supplementary Table 2.

Supplementary Table 3.

Supplementary Table 4.

Supplementary Table 5.

Supplementary Table 6.

Supplementary Table 7.

Supplementary Table 8.

Supplementary Table 9.

Supplementary Table 10.

Supplementary Table 11.

Supplementary Table 12.

Supplementary Table 13.

Supplementary Table 14.

Supplementary Table 15.

## Data Availability

The dataset analyzed during the current study is available in DepMap database version 21Q1 with the name CCLE_expression.csv. The sample code and data are publicly available at GitHub: https://github.com/DanielaMaresQ/WGCNA_BC_Cell.L.

## References

[1] World Health Organization. *Breast cancer*, accessed 12 April 2021; https://www.who.int/news-room/fact-sheets/detail/breast-cancer (2021).

[2] Henderson Craig I (2015). Breast Cancer Fundamentals of Evidence-Based Disease Management.

[3] Dunnwald LK, Rossing MA, Li CI (2007). Hormone receptor status, tumor characteristics, and prognosis: A prospective cohort of breast cancer patients. Breast Cancer Res..

[4] Harbeck N (2019). Breast cancer. Nat. Rev. Dis. Primers.

[5] Lüönd F, Tiede S, Christofori G (2021). Breast cancer as an example of tumour heterogeneity and tumour cell plasticity during malignant progression. Br. J. Cancer..

[6] Mei J, Zhao J, Fu Y (2020). Molecular classification of breast cancer using the mRNA expression profiles of immune-related genes. Sci. Rep..

[7] Tomlins SA (2015). Characterization of 1577 primary prostate cancers reveals novel biological and clinicopathologic insights into molecular subtypes. Eur. Urol..

[8] Hamam R (2017). Circulating microRNAs in breast cancer: Novel diagnostic and prognostic biomarkers. Cell Death Dis..

[9] Mautner BD, Schmidt KV, Brennan MB (2000). New diagnostic techniques and treatments for early breast cancer. Semin. Oncol. Nurs..

[10] Waks AG, Winer EP (2019). Breast cancer treatment: A review. JAMA.

[11] Peng L, Xu T, Long T, Zuo H (2016). Association between BRCA status and P53 status in breast cancer: A meta-analysis. Med. Sci. Monit..

[12] Dees S, Pontiggia L, Jasmin J-F, Mercier I (2020). Phosphorylated STAT3 (Tyr705) as a biomarker of response to pimozide treatment in triple-negative breast cancer. Cancer Biol. Ther..

[13] Brett JO, Spring LM, Bardia A, Wander SA (2021). ESR1 mutation as an emerging clinical biomarker in metastatic hormone receptor-positive breast cancer. Breast Cancer Res..

[14] Gam L-H (2012). Breast cancer and protein biomarkers. World J. Exp. Med..

[15] Adhami M, MotieGhader H, Haghdoost AA, Afshar RM, Sadeghi B (2020). Gene co-expression network approach for predicting prognostic microRNA biomarkers in different subtypes of breast cancer. Genomics.

[16] Liu Z, Li M, Hua Q, Li Y, Wang G (2019). Identification of an eight-lncRNA prognostic model for breast cancer using WGCNA network analysis and a Cox? Proportional hazards model based on L1-penalized estimation. Int. J. Mol. Med..

[17] Sideris N, Dama P, Bayraktar S, Stiff T, Castellano L (2022). LncRNAs in breast cancer: A link to future approaches. Cancer Gene Ther..

[18] Fachal, L. *et al*. Fine-mapping of 150 breast cancer risk regions identifies 178 high confidence target genes. 10.1101/521054v1 (2019).

[19] Zhang H (2019). Integrated analysis of oncogenic networks in colorectal cancer identifies GUCA2A as a molecular marker. Biochem. Res. Int..

[20] Fachal L (2020). Fine-mapping of 150 breast cancer risk regions identifies 191 likely target genes. Nat. Genet..

[21] Zugazagoitia J (2016). Current challenges in cancer treatment. Clin. Ther..

[22] Li M (2018). Transmembrane protein 170B is a novel breast tumorigenesis suppressor gene that inhibits the Wnt/β-catenin pathway. Cell Death Dis..

[23] Meurer SK, Tezcan O, Lammers T, Weiskirchen R (2020). Differential regulation of Lipocalin 2 (LCN2) in doxorubicin-resistant 4T1 triple negative breast cancer cells. Cell. Signal.

[24] Charan M (2020). Macrophage migration inhibitory factor inhibition as a novel therapeutic approach against triple-negative breast cancer. Cell Death Dis..

[25] Egusquiaguirre SP, Yeh JE, Walker SR, Liu S, Frank DA (2018). The STAT3 target gene TNFRSF1A modulates the NF-κB pathway in breast Cancer cells. Neoplasia.

[26] Yerlikaya A, Okur E, Ulukaya E (2012). The p53-independent induction of apoptosis in breast cancer cells in response to proteasome inhibitor bortezomib. Tumour Biol..

[27] de Amorim ÍSS (2019). Opposite effects of demethylating treatment on VEGFA and HIF1A expression in MDA-MB-231 breast cancer cell line in hypoxic microenvironment. Gene Rep..

[28] Dustin D (2021). RON signalling promotes therapeutic resistance in ESR1 mutant breast cancer. Br. J. Cancer..

[29] Amgalan D (2020). A small-molecule allosteric inhibitor of BAX protects against doxorubicin-induced cardiomyopathy. Nat. Cancer..

[30] Zhuang S, Li L, Zang Y, Li G, Wang F (2020). RRM2 elicits the metastatic potential of breast cancer cells by regulating cell invasion, migration and VEGF expression via the PI3K/AKT signaling. Oncol. Lett..

[31] Wang K (2020). Knockdown of MMP-1 inhibits the progression of colorectal cancer by suppressing the PI3K/Akt/c-myc signaling pathway and EMT. Oncol. Rep..

[32] Safran M, Abugessaisa I, Kasukawa T (2021). The gene cards suite. Practical Guide to Life Science Databases.

[33] Cox B, Sneyd M (2013). Bias in breast cancer research in the screening era. Breast.

[34] Renfro LA, An MW, Mandrekar SJ (2017). Precision oncology: A new era of cancer clinical trials. Cancer Lett..

[35] Marisa L (2013). Gene expression classification of colon cancer into molecular subtypes: Characterization, validation, and prognostic value. PLoS Med..

[36] Liu Z, Bai Y, Xie F, Miao F, Du F (2020). Comprehensive analysis for identifying diagnostic and prognostic biomarkers in colon adenocarcinoma. DNA Cell Biol..

[37] Shi Z, Derow CK, Zhang B (2010). Co-expression module analysis reveals biological processes, genomic gain, and regulatory mechanisms associated with breast cancer progression. BMC Syst. Biol..

[38] Abu N (2015). In vivo antitumor and antimetastatic effects of flavokawain B in 4T1 breast cancer cell-challenged mice. Drug Des. Dev. Ther..

[39] Liu Z, Li M, Fang X, Shen L, Yao W (2018). Identification of surrogate prognostic biomarkers for allergic asthma in nasal epithelial brushing samples by WGCNA. J. Cell. Biochem..

[40] Zheng PF, Chen LZ, Guan YZ, Liu P (2021). Weighted gene co-expression network analysis identifies specific modules and hub genes related to coronary artery disease. Sci. Rep..

[41] Bao C (2019). Exploring specific prognostic biomarkers in triple-negative breast cancer. Cell Death Dis..

[42] Bettaieb A (2017). Precision medicine in breast cancer: Reality or utopia?. J. Transl. Med..

[43] Ghandi M (2019). Next-generation characterization of the cancer cell line encyclopedia. Nature.

[44] Broad DepMap. *DepMap 21Q1 Public (p. 12817867672 Bytes)*. figshare. 10.6084/M9.FIGSHARE.13681534.V2 (2021).

[45] Langfelder P, Horvath S (2008). WGCNA: An R package for weighted correlation network analysis. BMC Bioinform..

[46] Langfelder P, Zhang B, Horvath S (2008). Defining clusters from a hierarchical cluster tree: The dynamic tree cut package for R. Bioinformatics.

[47] Horvath S (2011). Weighted Network Analysis: Applications in Genomics and Systems Biology.

[48] Kanehisa M, Goto S (2000). KEGG: Kyoto encyclopedia of genes and genomes. Nucleic Acids Res..

[49] Kanehisa M, Furumichi M, Sato Y, Kawashima M, Ishiguro-Watanabe M (2023). KEGG for taxonomy-based analysis of pathways and genomes. Nucleic Acids Res..

[50] Kanehisa M (2019). Toward understanding the origin and evolution of cellular organisms. Protein Sci..

[51] Lee S (2020). Landscape analysis of adjacent gene rearrangements reveals BCL2L14-ETV6 gene fusions in more aggressive triple-negative breast cancer. Proc. Natl. Acad. Sci..

[52] Kammerer S (2016). KCNJ3 is a new independent prognostic marker for estrogen receptor positive breast cancer patients. Oncotarget.

[53] Pavón L, del Carmen Jiménez M, Garcés ME (2020). Inmunología Molecular, Celular y Traslacional.

[54] Goff SL, Danforth DN (2021). The role of immune cells in breast tissue and immunotherapy for the treatment of breast cancer. Clin. Breast Cancer.

[55] Landskron G, De la Fuente M, Thuwajit P, Thuwajit C, Hermoso MA (2014). Chronic inflammation and cytokines in the tumor microenvironment. J. Immunol. Res..

[56] Xiong Y (2022). High expression of CD58 and ALDH1A3 predicts a poor prognosis in basal-like breast cancer. Anticancer Res..

[57] Banerji S (2012). Sequence analysis of mutations and translocations across breast cancer subtypes. Nature.

[58] Clark AG, Vignjevic DM (2015). Modes of cancer cell invasion and the role of the microenvironment. Curr. Opin. Cell Biol..

[59] Kennecke H (2010). Metastatic behavior of breast cancer subtypes. J. Clin. Oncol..

[60] Du Y, Wang P (2019). Upregulation of MIIP regulates human breast cancer proliferation, invasion and migration by mediated by IGFBP2. Pathol. Res. Pract..

[61] Wang N (2018). CXCL1 derived from tumor-associated macrophages promotes breast cancer metastasis via activating NF-κB/SOX4 signaling. Cell Death Dis..

[62] Kaur P (2021). Identification of putative actionable alterations in clinically relevant genes in breast cancer. Br. J. Cancer.

[63] Choromanska A (2021). Modifications of plasma membrane organization in cancer cells for targeted therapy. Molecules.

[64] Padua MB (2018). Dependence receptor UNC5A restricts luminal to basal breast cancer plasticity and metastasis. Breast Cancer Res..

[65] Arnal J-F (2017). Membrane and nuclear estrogen receptor alpha actions: From tissue specificity to medical implications. Physiol. Rev..

[66] Eroles P, Bosch A, Pérez-Fidalgo JA, Lluch A (2012). Molecular biology in breast cancer: Intrinsic subtypes and signaling pathways. Cancer Treat. Rev..

[67] Santarpia L (2016). Deciphering and targeting oncogenic mutations and pathways in breast cancer. Oncologist.

[68] Gao J, Swain S (2018). Luminal a breast cancer and molecular assays: A review. Oncologist.

[69] Mayayo-Peralta I, Prekovic S, Zwart W (2021). Estrogen receptor on the move: Cistromic plasticity and its implications in breast cancer. Mol Aspects Med..

[70] Xiong GF, Xu R (2016). Function of cancer cell-derived extracellular matrix in tumor progression. JCMT.

[71] Tan Q (2023). Breast cancer cells interact with tumor-derived extracellular matrix in a molecular subtype-specific manner. Biomater. Adv..

[72] Diaz Bessone MI, Gattas MJ, Laporte T, Tanaka M, Simian M (2019). The tumor microenvironment as a regulator of endocrine resistance in breast cancer. Front. Endocrinol..

[73] Cheang M (2009). Ki67 index, HER2 status, and prognosis of patients with luminal B breast cancer. J. Natl. Cancer Inst..

[74] Toy W (2017). Activating ESR1 mutations differentially affect the efficacy of ER antagonists. Cancer Discov..

[75] Thapa M, Dallmann G (2020). Role of coenzymes in cancer metabolism. Semin. Cell Dev. Biol..

[76] Moasser MM (2007). The oncogene HER2: Its signaling and transforming functions and its role in human cancer pathogenesis. Oncogene.

[77] Razavi P (2018). The genomic landscape of endocrine-resistant advanced breast cancers. Cancer Cell..

[78] Duffy MJ, Synnott NC, Crown J (2018). Mutant p53 in breast cancer: Potential as a therapeutic target and biomarker. Breast Cancer Res. Treat..

[79] Kong X (2019). Mechanism of trastuzumab resistance caused by HER-2 mutation in breast carcinomas. Cancer Manag. Res..

[80] Farahmand M (2019). Epstein–Barr virus and risk of breast cancer: A systematic review and meta-analysis. Future Oncol..

[81] Gupta I (2021). Presence of high-risk HPVs, EBV, and MMTV in human triple-negative breast cancer. Hum. Vaccines Immunother..

[82] Ingerslev K (2019). The prevalence of EBV and CMV DNA in epithelial ovarian cancer. Infect. Agents Cancer.

[83] Ahrens TD (2020). The role of proteoglycans in cancer metastasis and circulating tumor cell analysis. Front. Cell Dev. Biol..

[84] Gwenaëlle Lemoine. *GWENA: Pipeline for augmented co-expression analysis*. (R package Version 1.0.1); https://rdrr.io/bioc/GWENA/man/get_hub_degree.html (2021).

[85] Li X, Sun G, Wu L, Sun G, Cheng Y (2021). Upregulation of ADAR promotes breast cancer progression and serves as a potential therapeutic target. J. Oncol..

[86] Pidugu VK, Pidugu HB, Wu MM, Liu CJ, Lee TC (2019). Emerging functions of human IFIT proteins in cancer. Front. Mol. Biosci..

[87] Khazei K (2022). Transcriptome profiling of curcumin-treated T47D human breast cancer cells by a system-based approach. Gene Rep..

[88] Lamsal A (2023). Opposite and dynamic regulation of the interferon response in metastatic and non-metastatic breast cancer. J. Cell Commun. Signal.

[89] Rouette A (2016). Expression of immunoproteasome genes is regulated by cell-intrinsic and -extrinsic factors in human cancers. Sci. Rep..

[90] Flick K, Kaiser P (2012). Protein degradation and the stress response. Semin. Cell Dev. Biol..

[91] Maia Falcão R (2022). The expression of the immunoproteasome subunit PSMB9 is related to distinct molecular subtypes of uterine leiomyosarcoma. Cancers.

[92] Sherman BT (2022). DAVID: A web server for functional enrichment analysis and functional annotation of gene lists (2021 update). Nucleic Acids Res..

[93] Thomas PD (2003). PANTHER: A browsable database of gene products organized by biological function, using curated protein family and subfamily classification. Nucleic Acids Res..

[94] Perou CM (2000). Molecular portraits of human breast tumours. Nature.

[95] Wu M (2017). Targeting ETS1 with RNAi-based supramolecular nanoassemblies for multidrug-resistant breast cancer therapy. J. Control Release.

[96] Mei D (2021). Microarray profile analysis identifies ETS1 as potential biomarker regulated by miR-23b and modulates TCF4 in gastric cancer. World J. Surg. Oncol..

[97] Li Y, Wu T, Peng Z, Tian X, Dai Q (2022). ETS1 is a prognostic biomarker of triple-negative breast cancer and promotes the triple-negative breast cancer progression through the YAP signaling. Am. J. Cancer Res..

[98] Cal S, López-Otín C (2015). ADAMTS proteases and cancer. Matrix Biol..

[99] Xie Y (2016). ADAMTS6 suppresses tumor progression via the ERK signaling pathway and serves as a prognostic marker in human breast cancer. Oncotarget.

[100] Li J, Xu Q, Wang W, Sun S (2019). Biosci. Rep..

[101] Crudele F (2020). The network of non-coding RNAs and their molecular targets in breast cancer. Mol. Cancer.

[102] Alkhateeb A (2019). Transcriptomics signature from next-generation sequencing data reveals new transcriptomic biomarkers related to prostate cancer. Cancer Inform..

[103] Qian X-L (2019). Caveolin-1: A multifaceted driver of breast cancer progression and its application in clinical treatment. Onco Targets Ther..

[104] Erdemli HK (2016). Is serum caveolin-1 a useful biomarker for progression in patients with colorectal cancer. Clin Lab.

[105] Gumulec J (2012). Caveolin-1 as a potential high-risk prostate cancer biomarker. Oncol. Rep..

[106] Leiser D (2021). Role of caveolin-1 as a biomarker for radiation resistance and tumor aggression in lung cancer. PLoS ONE.

[107] Mahmood J, Murti SC, Zaveri SR, Shukla HD, Vujaskovic Z (2016). Caveolin-1: A novel prognostic biomarker for radioresistance in non-small cell lung carcinoma (NSCLC) and prostate cancer. IJROBP.

[108] Panic A (2021). The biomarker potential of Caveolin-1 in penile cancer. Front. Oncol..

[109] Al Abo M (2022). Adaptive stress response genes associated with breast cancer subtypes and survival outcomes reveal race-related differences. NPJ Breast Cancer.

[110] Yates AD (2020). Ensembl 2020. Nucleic Acids Res..

[111] Zhang Y (2016). Long noncoding RNA LINP1 regulates repair of DNA double-strand breaks in triple-negative breast cancer. Nat. Struct. Mol. Biol..

[112] Xue Y (2022). Database resources of the national genomics data center, China National Center for Bioinformation in 2022. Nucleic Acids Res..

[113] Xu Y (2020). Carbonic anhydrase 4 serves as a clinicopathological biomarker for outcomes and immune infiltration in renal cell carcinoma, lower grade glioma, lung adenocarcinoma and uveal melanoma. J. Cancer..

[114] Wang B (2020). Carbonic anhydrase IV inhibits cell proliferation in gastric cancer by regulating the cell cycle. Oncol. Lett..

[115] Liu L, Zhang Y, Lu J (2020). The roles of long noncoding RNAs in breast cancer metastasis. Cell Death Dis..

[116] Liu C, Hu C, Li J, Jiang L, Zhao C (2021). Identification of epithelial-mesenchymal transition-related lncRNAs that associated with the prognosis and immune microenvironment in colorectal cancer. Front. Mol. Biosci..

[117] Liu S (2021). A novel lncRNA ROPM-mediated lipid metabolism governs breast cancer stem cell properties. J. Hematol. Oncol..

[118] Liu Y, Tingart M, Lecouturier S, Li J, Eschweiler J (2021). Identification of co-expression network correlated with different periods of adipogenic and osteogenic differentiation of BMSCs by weighted gene co-expression network analysis (WGCNA). BMC Genom..

[119] Su R (2021). Construction of a ceRNA network of hub genes affecting immune infiltration in ovarian cancer identified by WGCNA. BMC Cancer.

[120] Fang Y, Yu H, Zhou H (2022). MS4A15 acts as an oncogene in ovarian cancer through reprogramming energy metabolism. Biochem. Biophys. Res. Commun..

[121] Sun L, Zhang Y, Zhang C (2018). Distinct expression and prognostic value of MS4A in gastric cancer. Open Med..

[122] Liu C (2021). Upregulation of LINC00511 expression by DNA hypomethylation promotes the progression of breast cancer. Gland Surg..

[123] Lo PHY, Tanikawa C, Katagiri T, Nakamura Y, Matsuda K (2015). Identification of novel epigenetically inactivated gene PAMR1 in breast carcinoma. Oncol. Rep..

[124] Yang R (2021). High expression of PAMR1 predicts favorable prognosis and inhibits proliferation, invasion, and migration in cervical cancer. Front. Oncol..

[125] Lee C (2019). Transthyretin stimulates tumor growth through regulation of tumor. Immune Endothel. Cells. J. Immunol..

[126] Sharma M, Khan S, Rahman S, Singh LR (2019). The extracellular protein, transthyretin is an oxidative stress biomarker. Front. Physiol..

[127] Ingenbleek Y (2022). Plasma transthyretin is a nutritional biomarker in human morbidities. Front. Med..

[128] Zhang H (2021). Exploration of novel candidate genes involved in epidermal keratinocyte differentiation and skin barrier repair in man. Differentiation.

[129] Schettini F (2021). Identification of cell surface targets for CAR-T cell therapies and antibody-drug conjugates in breast cancer. ESMO Open.

[130] Goll DE, Thompson VF, Li H, Wei W, Cong J (2003). The calpain system. Physiol. Rev..

[131] Davis J (2014). Low calpain-9 is associated with adverse disease-specific survival following endocrine therapy in breast cancer. BMC Cancer.

[132] Vergara Bermejo A, Ragonnaud E, Daradoumis J, Holst P (2020). Cancer associated endogenous retroviruses: Ideal immune targets for adenovirus-based immunotherapy. Int. J. Mol. Sci..

[133] Wang-Johanning F (2014). Human endogenous retrovirus type K antibodies and mRNA as serum biomarkers of early-stage breast cancer. Int. J. Cancer.

[134] Talaat IM (2020). Bone marrow mammaglobin-1 (SCGB2A2) immunohistochemistry expression as a breast cancer specific marker for early detection of bone marrow micrometastases. Sci. Rep..

[135] Watson MA, Darrow C, Zimonjic DB, Popescu NC, Fleming TP (1998). Structure and transcriptional regulation of the human mammaglobin gene, a breast cancer associated member of the uteroglobin gene family localized to chromosome 11q13. Oncogene.

[136] Span PN (2004). Mammaglobin is associated with low-grade, steroid receptor-positive breast tumors from postmenopausal patients, and has independent prognostic value for relapse-free survival time. J. Clin. Oncol..

[137] Zhang K (2022). Downregulated expression of RIPOR3 correlated with immune infiltrates predicts poor prognosis in oral tongue cancer. Med. Sci. Monit..

[138] Albert M (2022). ISG15 Is a novel regulator of lipid metabolism during vaccinia virus infection. Microbiol. Spectr..

[139] Han HG, Moon HW, Jeon YJ (2018). ISG15 in cancer: Beyond ubiquitin-like protein. Cancer Lett..

[140] Lee K, Hong HR, Lim JS, Ko KP, Lee MG (2022). XAF1 drives apoptotic switch of endoplasmic reticulum stress response through destabilization of GRP78 and CHIP. Cell Death Dis..

[141] Tran V (2020). Influenza virus repurposes the antiviral protein IFIT2 to promote translation of viral mRNAs. Nat. Microbiol..

[142] Gu X (2016). Epigenetic regulation of OAS2 shows disease-specific DNA methylation profiles at individual CpG sites. Sci. Rep..

[143] Senbanjo LT, Chellaiah MA (2017). CD44: A multifunctional cell surface adhesion receptor is a regulator of progression and metastasis of cancer cells. Front. Cell Dev. Biol..

[144] Fang L (2021). PLAU directs conversion of fibroblasts to inflammatory cancer-associated fibroblasts, promoting esophageal squamous cell carcinoma progression via uPAR/Akt/NF-κB/IL8 pathway. Cell Death Discov..

[145] Nilsson UW, Abrahamsson A, Dabrosin C (2010). Angiogenin regulation by estradiol in breast tissue: Tamoxifen inhibits angiogenin nuclear translocation and antiangiogenin therapy reduces breast cancer growth in vivo. Clin. Cancer Res..

[146] Chen L, Xiao D, Tang F, Gao H, Li X (2020). CAPN6 in disease: An emerging therapeutic target (Review). Int. J. Mol. Med..

[147] Magalhães J, Eira J, Liz MA (2021). The role of transthyretin in cell biology: Impact on human pathophysiology. Cell Mol. Life Sci..

[148] Pittois K, Wauters J, Bossuyt P, Deleersnijder W, Merregaert J (1999). Genomic organization and chromosomal localization of the Itm2a gene. Mamm. Genome.

[149] Aurilio G (2020). Androgen receptor signaling pathway in prostate cancer: From genetics to clinical applications. Cells.

[150] Sjöstedt E (2020). An atlas of the protein-coding genes in the human, pig, and mouse brain. Science.

[151] Weichselbaum RR (2008). An interferon-related gene signature for DNA damage resistance is a predictive marker for chemotherapy and radiation for breast cancer. Proc. Natl. Acad. Sci. USA.

[152] Nushtaeva AA (2018). Characterization of primary normal and malignant breast cancer cell and their response to chemotherapy and immunostimulatory agents. BMC Cancer..

[153] Yu DH (2020). Effects of hub genes on the clinicopathological and prognostic features of lung adenocarcinoma. Oncol. Lett..

[154] Uhlen M (2017). A pathology atlas of the human cancer transcriptome. Science.

[155] Charafe-Jauffret E (2006). Gene expression profiling of breast cell lines identifies potential new basal markers. Oncogene.

[156] Calderón-González K (2015). Determination of the protein expression profiles of breast cancer cell lines by quantitative proteomics using iTRAQ labelling and tandem mass spectrometry. J. Proteom..

[157] Stein T (2005). Annexin A8 is up-regulated during mouse mammary gland involution and predicts poor survival in breast cancer. Clin. Cancer Res..

[158] Muthamilselvan S, Palaniappan A (2023). Brcadx: Precise identification of breast cancer from expression data using a minimal set of features. Front. Bioinform..

[159] Wu J, Liu XJ, Hu JN, Liao XH, Lin FF (2020). Transcriptomics and prognosis analysis to identify critical biomarkers in invasive breast carcinoma. Technol. Cancer Res. Treat..

[160] Jiang G (2016). Comprehensive comparison of molecular portraits between cell lines and tumors in breast cancer. BMC Genom..

[161] Gambardella G (2022). A single-cell analysis of breast cancer cell lines to study tumour heterogeneity and drug response. Nat. Commun..

[162] Liu K (2019). Evaluating cell lines as models for metastatic breast cancer through integrative analysis of genomic data. Nat. Commun..

