# Supplementary material for: Identification of modules and key genes associated with breast cancer subtypes through network analysis
Source: Sci Rep. 2024 May 29;14:12350. doi: 10.1038/s41598-024-61908-4 (PMC11137066; doi:10.1038/s41598-024-61908-4)
Supplement: Supplementary file 1 — Supplementary Information. [file 41598_2024_61908_MOESM1_ESM.docx]

**Supplementary Legends**

**Supplementary Figure Legends**Supplemental Figure S1. Determination of soft thresholding power in WGCNA. A)
Analysis of the scale-free fit index for various soft-thresholding powers. B) Analysis of the
mean connectivity for various soft-thresholding powers.
Supplemental Figure S2. Cluster of module eigengenes.
Supplemental Figure S3. Heatmap of the correlation between module eigengenes and
different subtypes of breast cancer.
Supplemental Figure S4. Protein-Protein Interaction Network for each SBC. In this network, the colors of the nodes represent various types of evidence or characteristics. Red nodes indicate gene fusion events. This implies that the proteins are either fused into a single gene or encoded by a fused gene; Blue nodes are proteins that tend to be present together in evolutionarily related organisms. Purple nodes are interactions that have been demonstrated in laboratory experiments. Yellow: Textual information from scientific literature supports these associations. Light Green: coexpression, these proteins tend to express themselves together in biological samples; Dark Green: these associations are derived from databases and annotation resources; Orange: these proteins are close to each other in the network context. (a) Protein-protein interaction network for the subtype Basal A. (b) Protein-protein interaction network for subtype Basal B. (c) Protein-protein interaction network for subtype Luminal A. (d) Protein-protein interaction network for subtype Luminal B. (e) Protein-protein interaction network for subtype Her2 ampl

**Supplementary Table Legends**

Supplemental Table S1. Characteristics of BC cell lines.
Supplemental Table S2. Threshold value for different 50 genes with higher co-expression of
SBC.

Supplemental Table S3. Functional Enrichment for Biological Process.
Supplemental Table S4. Functional Enrichment for Cellular Components.
Supplemental Table S5. Functional Enrichment for Molecular Functions.
Supplemental Table S6. Functional Enrichment for Pathways.
Supplemental Table S7. Top 50 highly degree genes for each of SBC.
Supplemental Table S8. Features of the five hub genes for each SBC.
Supplemental Table S9. Features of Basal A network.
Supplemental Table S10. Features of Basal B network.
Supplemental Table S11. Features of Luminal A network.
Supplemental Table S12. Features of Luminal B network.
Supplemental Table S13. Features of Her2ampl network.

Supplemental Table S14. Hub genes in patients for each breast cancer subtype.

Supplemental Table S15. Identification of our proposed genes as biomarkers in other breast cancer studies. (***) Genes are present in the top five hub genes of our module, (**) genes in the top 50 hub genes of our module and (*) genes are present throughout our module.
