# Supplementary figures and images for: Identification of modules and key genes associated with breast cancer subtypes through network analysis
Source: Sci Rep. 2024 May 29;14:12350. doi: 10.1038/s41598-024-61908-4 (PMC11137066; doi:10.1038/s41598-024-61908-4)

a

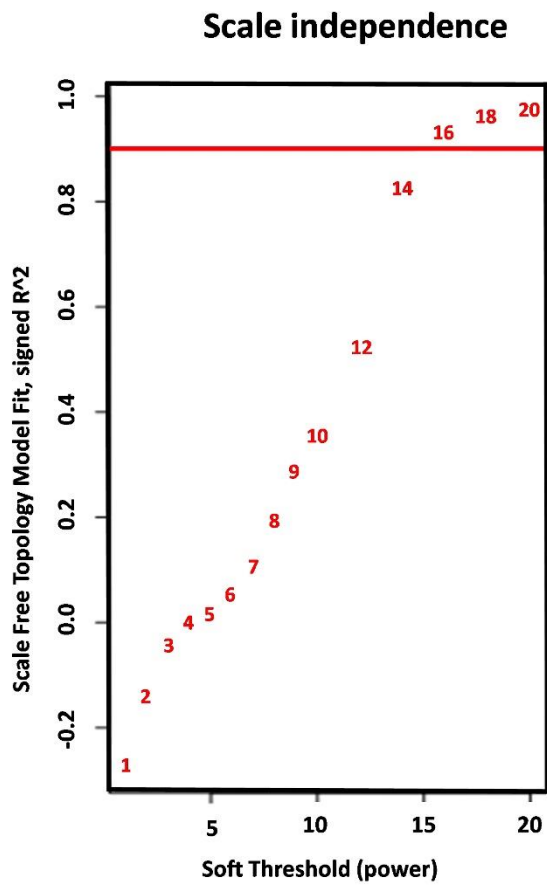

b

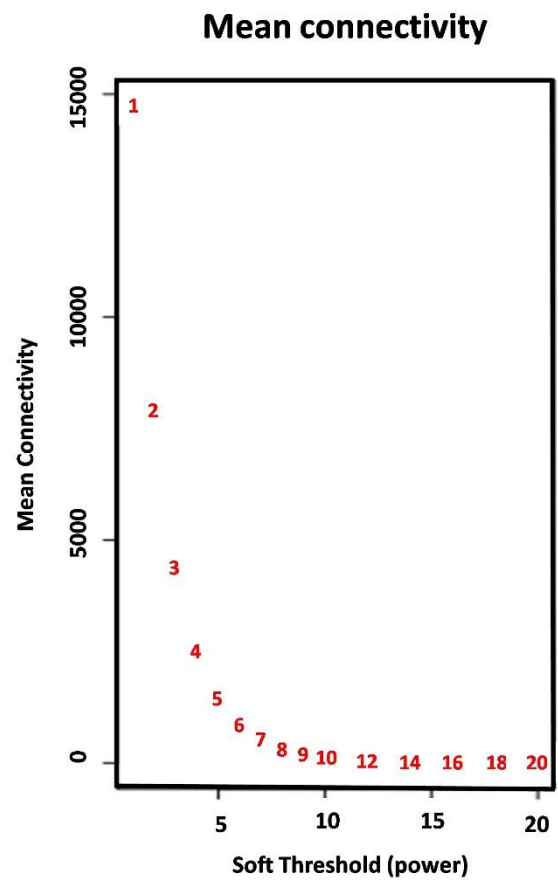

Supplement: Supplementary file 2 — Supplementary Figure 1. [file 41598_2024_61908_MOESM2_ESM.pdf]

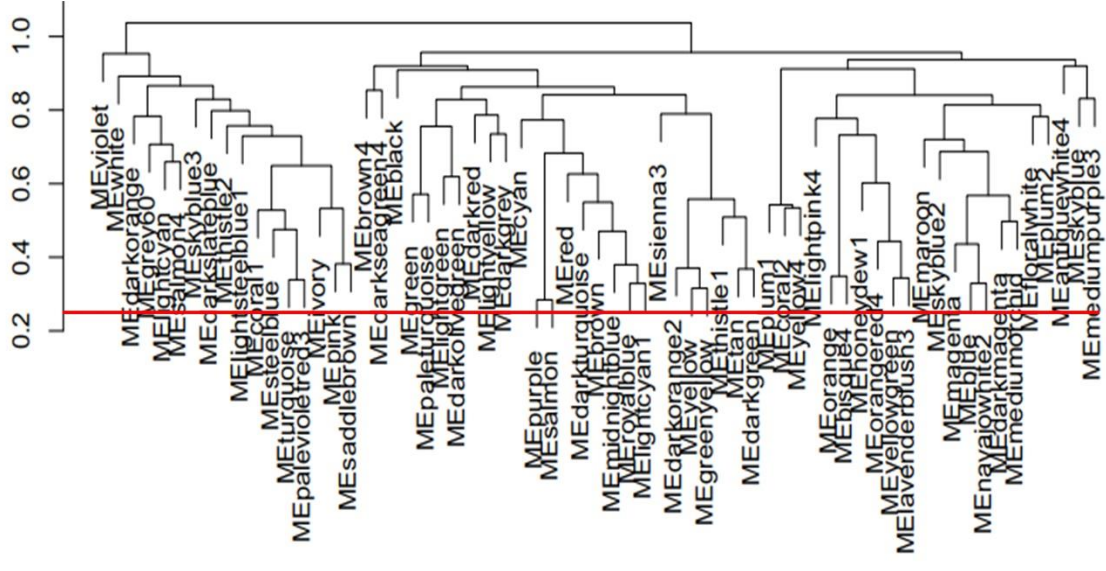

Supplement: Supplementary file 3 — Supplementary Figure 2. [file 41598_2024_61908_MOESM3_ESM.pdf]

**a**

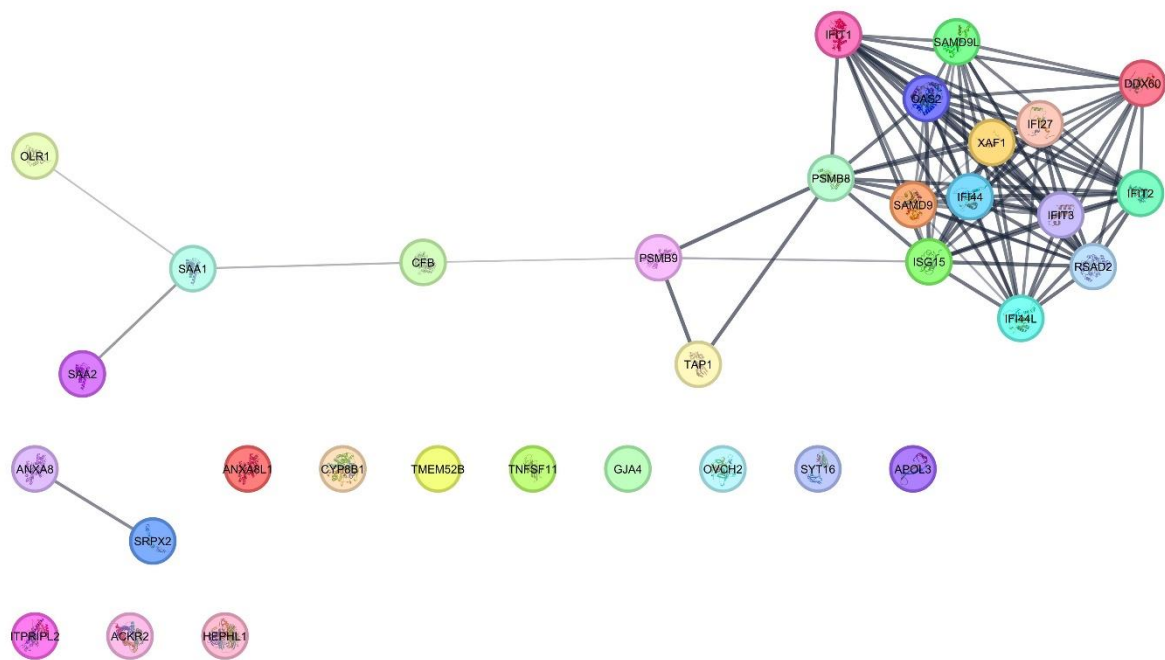

**b**

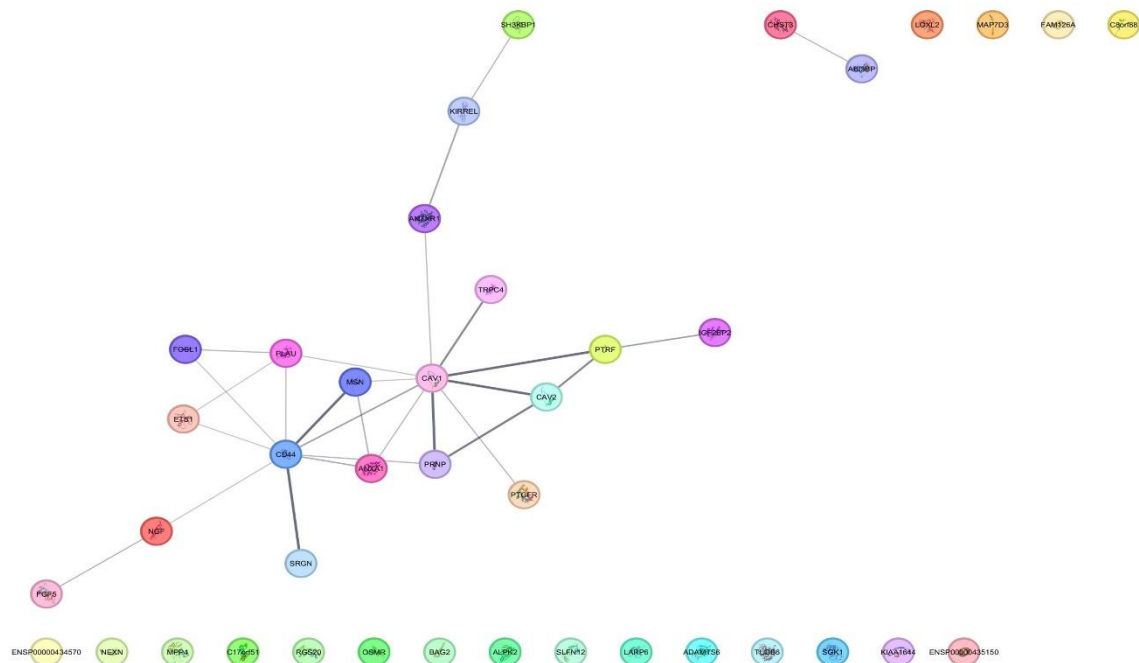

c

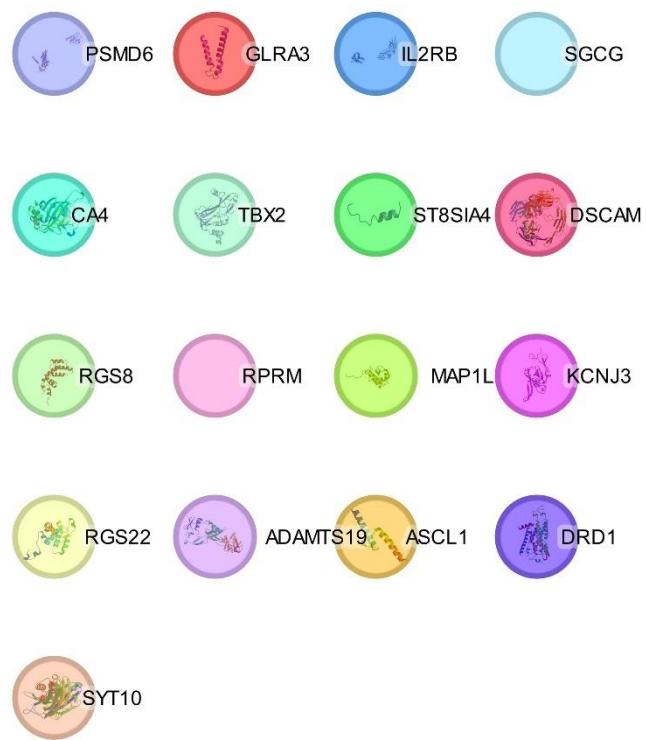

d

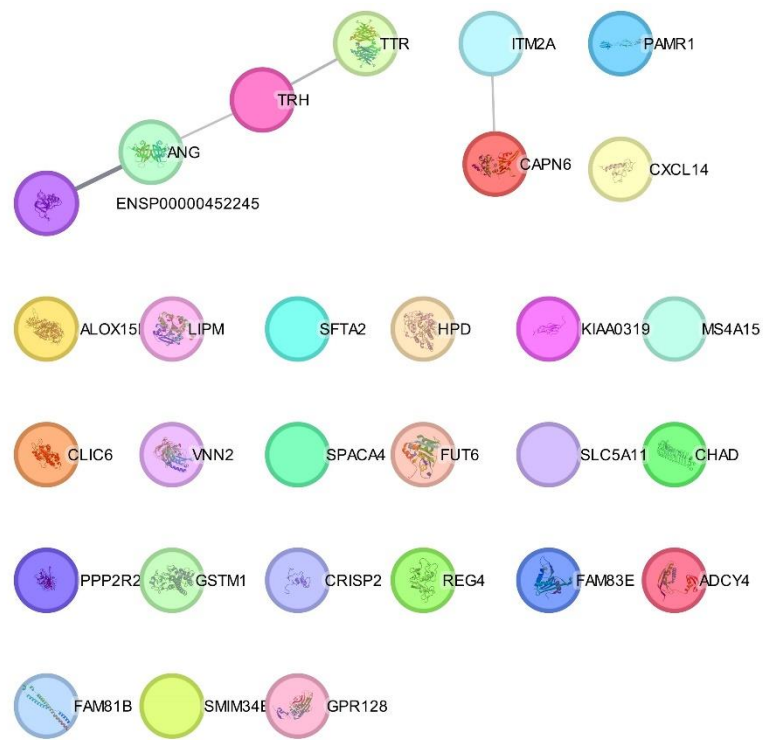

e

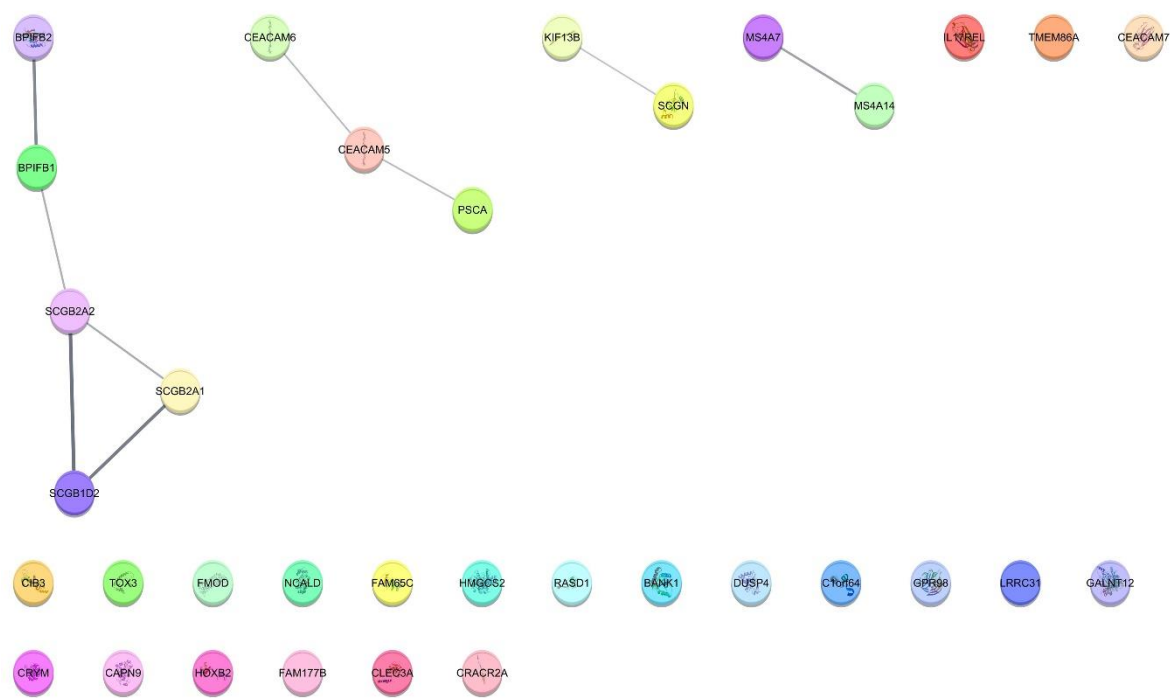

Supplement: Supplementary file 5 — Supplementary Figure 4. [file 41598_2024_61908_MOESM5_ESM.pdf]
