# Supplementary material for: Identification of modules and key genes associated with breast cancer subtypes through network analysis
Source: Sci Rep. 2024 May 29;14:12350. doi: 10.1038/s41598-024-61908-4 (PMC11137066; doi:10.1038/s41598-024-61908-4)

Module−trait relationships(page 1)

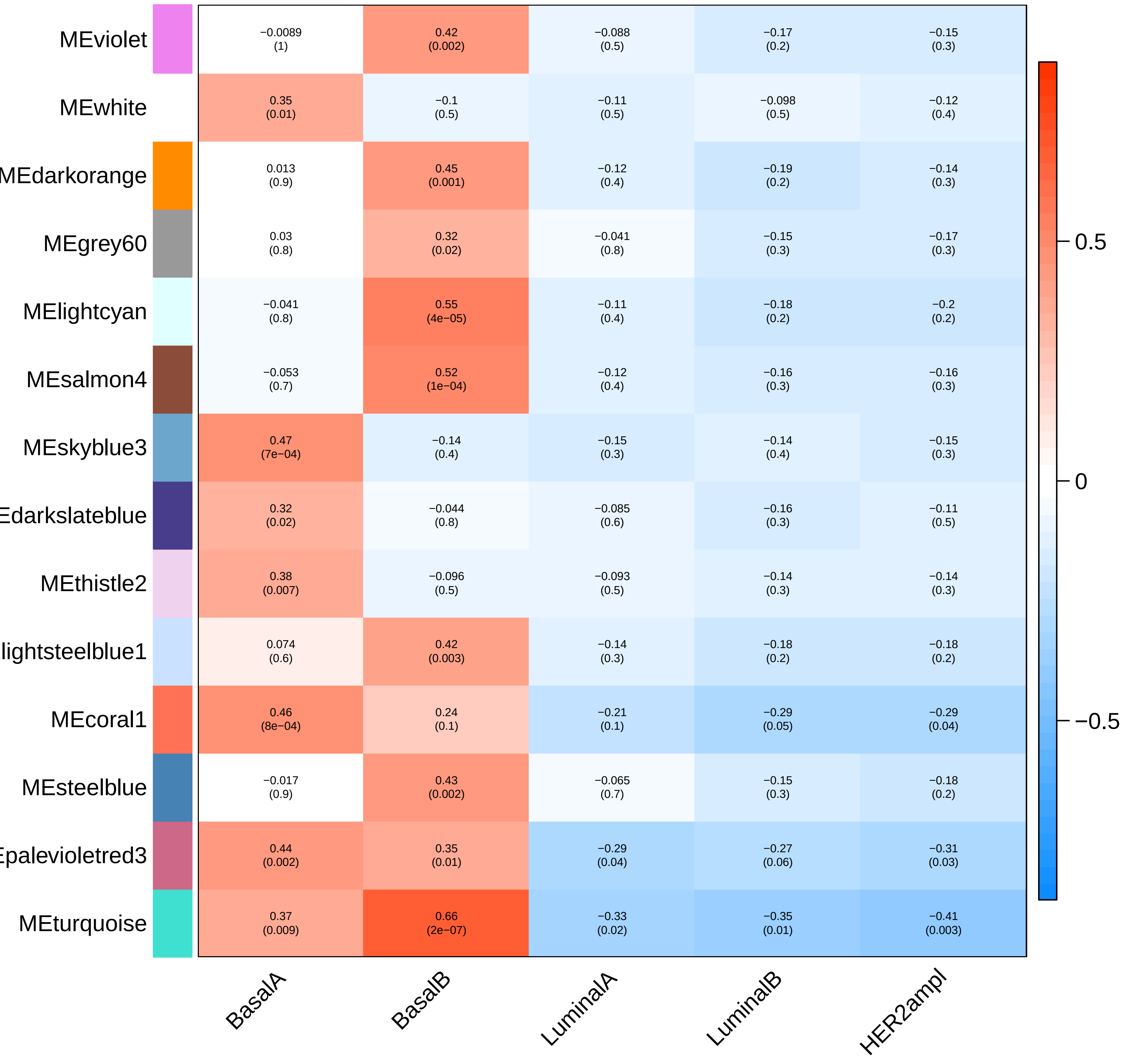

Module−trait relationships(page 2)

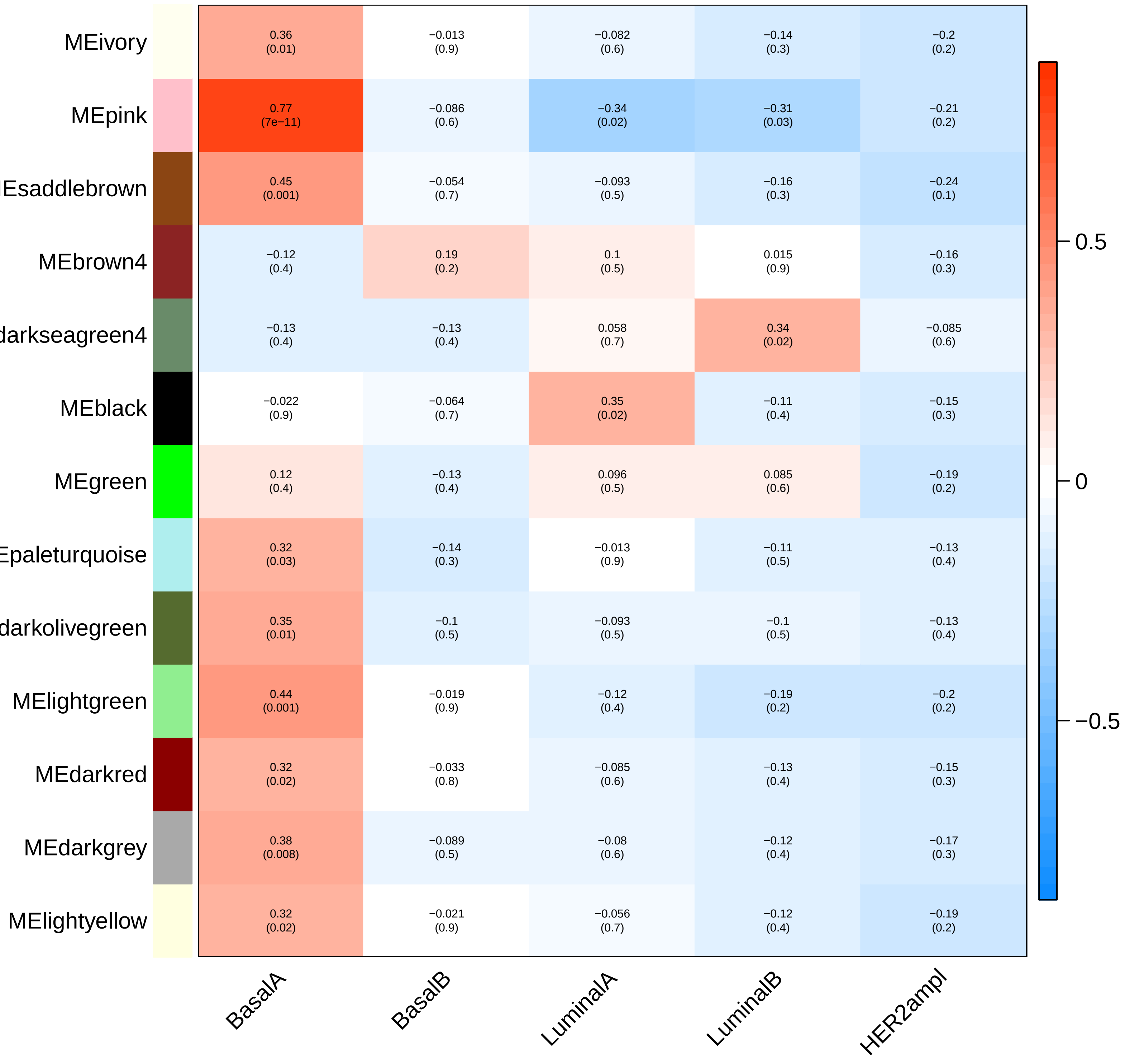

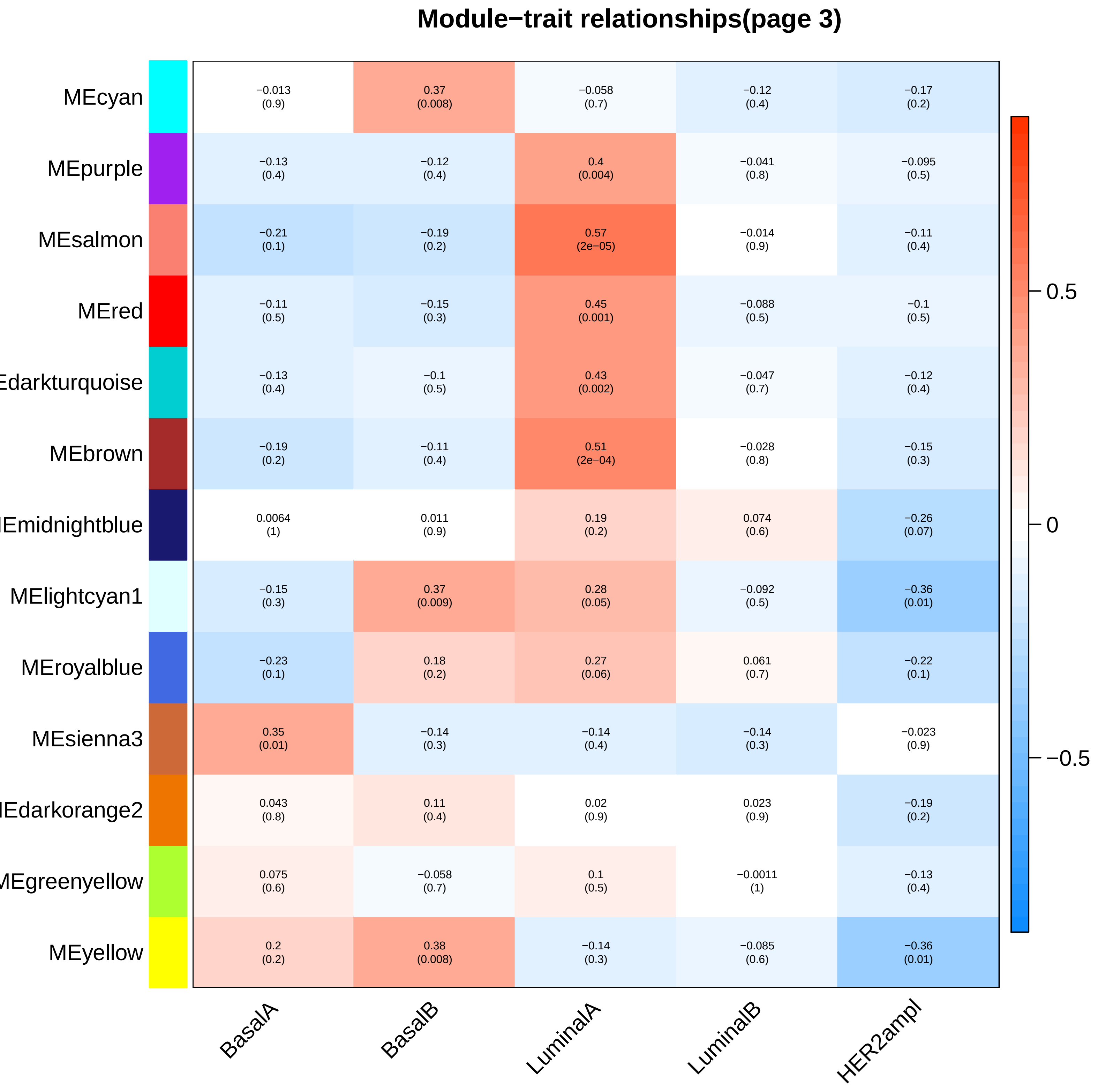

Module−trait relationships(page 3)
MEcyan
MEpurple
MEsalmon
MEred
Edarkturquoise
MEbrown
IEmidnightblue
MElightcyan1
MEroyalblue
MEsienna3
IEdarkorange2
MEgreenyellow
MEyellow
BasalA
BasalB
LuminalA
LuminalB
HER2ampl
−0.013 (0.9)
0.37 (0.008)
−0.058 (0.7)
−0.12 (0.4)
−0.17 (0.2)
−0.13 (0.4)
−0.12 (0.4)
0.4 (0.004)
−0.041 (0.8)
−0.095 (0.5)
−0.21 (0.1)
−0.19 (0.2)
0.57 (2e−05)
−0.014 (0.9)
−0.11 (0.4)
−0.11 (0.5)
−0.15 (0.3)
0.45 (0.001)
−0.088 (0.5)
−0.1 (0.5)
−0.13 (0.4)
−0.1 (0.5)
0.43 (0.002)
−0.047 (0.7)
−0.12 (0.4)
−0.19 (0.2)
−0.11 (0.4)
0.51 (2e−04)
−0.028 (0.8)
−0.15 (0.3)
0.0064 (1)
0.011 (0.9)
0.19 (0.2)
0.074 (0.6)
−0.26 (0.07)
−0.15 (0.3)
0.37 (0.009)
0.28 (0.05)
−0.092 (0.5)
−0.36 (0.01)
−0.23 (0.1)
0.18 (0.2)
0.27 (0.06)
0.061 (0.7)
−0.22 (0.1)
0.35 (0.01)
−0.14 (0.3)
−0.14 (0.4)
−0.14 (0.3)
−0.023 (0.9)
0.043 (0.8)
0.11 (0.4)
0.02 (0.9)
0.023 (0.9)
−0.19 (0.2)
0.075 (0.6)
−0.058 (0.7)
0.1 (0.5)
−0.0011 (1)
−0.13 (0.4)
0.2 (0.2)
0.38 (0.008)
−0.14 (0.3)
−0.085 (0.6)
−0.36 (0.01)
0.5
0
−0.5

Module−trait relationships(page 4)

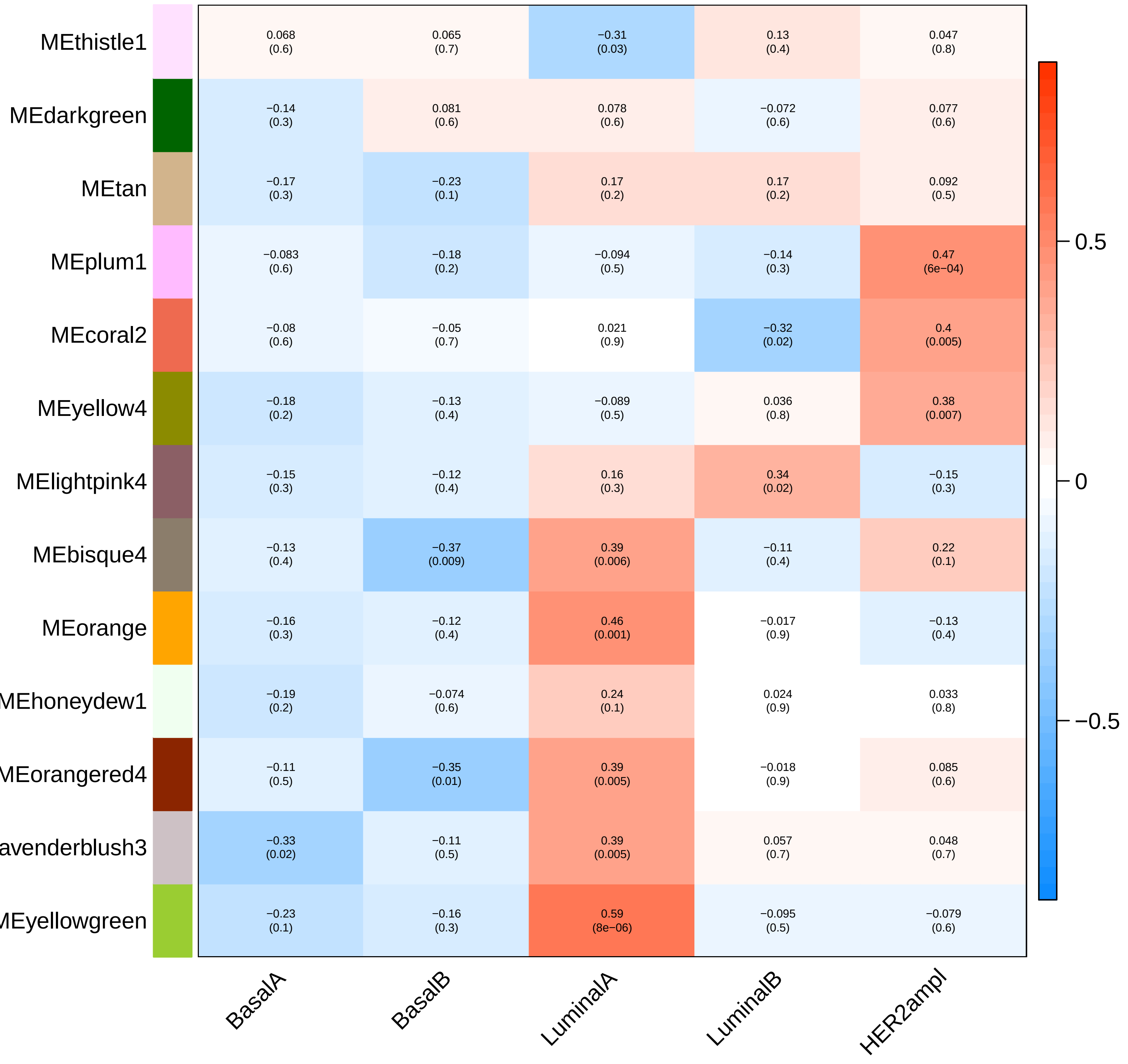

Module−trait relationships(page 5)

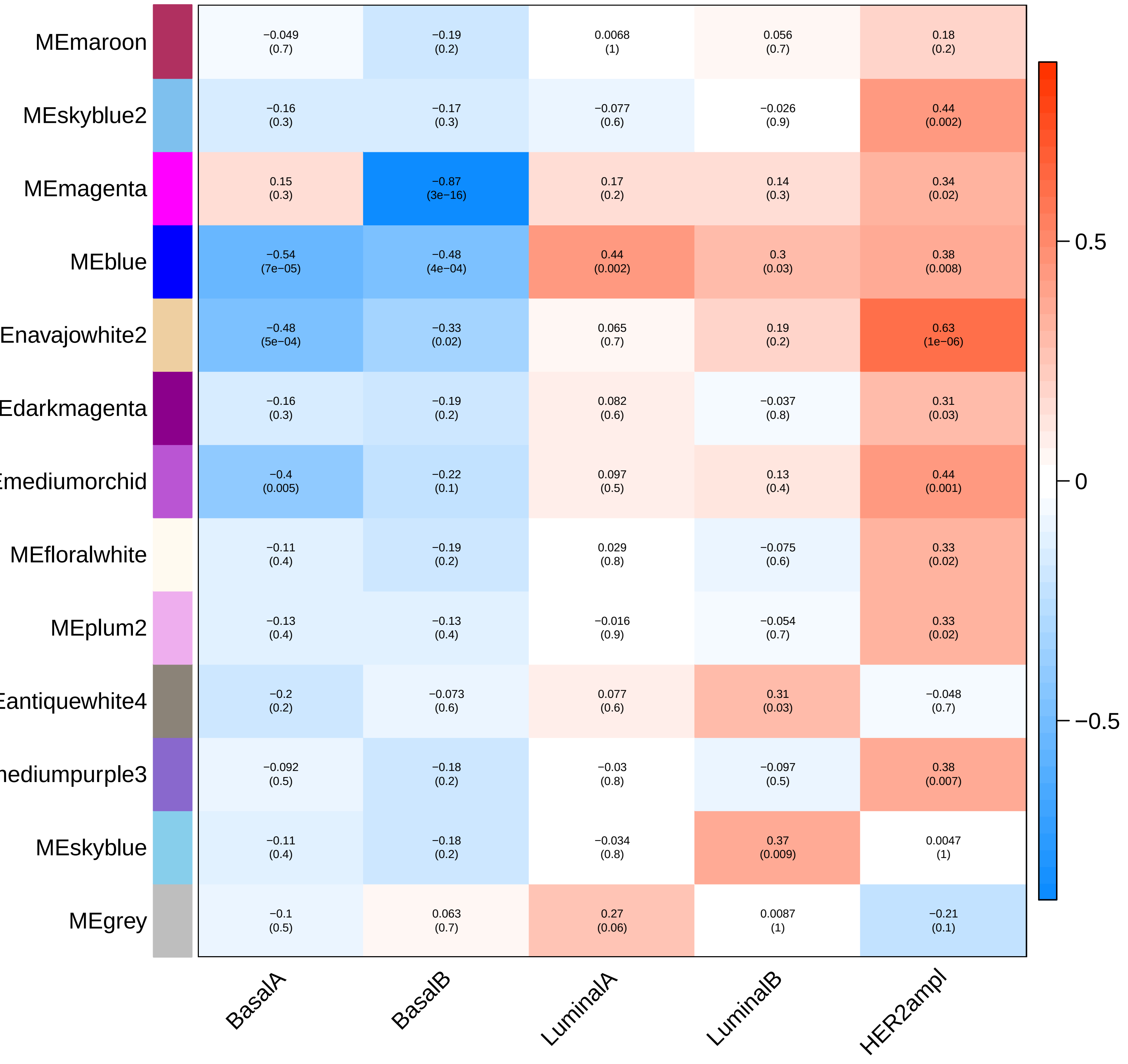

Supplement: Supplementary file 4 — Supplementary Figure 3. [file 41598_2024_61908_MOESM4_ESM.pdf]
